# Antimicrobial Treatment and Prophylaxis of Plague: Recommendations for Naturally Acquired Infections and Bioterrorism Response

**DOI:** 10.15585/mmwr.rr7003a1

**Published:** 2021-07-16

**Authors:** Christina A. Nelson, Dana Meaney-Delman, Shannon Fleck-Derderian, Katharine M. Cooley, Patricia A. Yu, Paul S. Mead

**Affiliations:** ^1^Bacterial Diseases Branch, Division of Vector-Borne Diseases, National Center for Emerging and Zoonotic Infectious Diseases, CDC, Fort Collins, Colorado; ^2^Infant Outcomes Monitoring, Research and Prevention Branch, Division of Birth Defects and Infant Disorders, National Center on Birth Defects and Developmental Disabilities, CDC, Atlanta, Georgia; ^3^Division of Preparedness and Emerging Infections, National Center for Emerging and Zoonotic Infectious Diseases, CDC, Atlanta, Georgia

## Abstract

This report provides CDC recommendations to U.S. health care providers regarding treatment, pre-exposure prophylaxis, and postexposure prophylaxis of plague. *Yersinia pestis*, the bacterium that causes plague, leads to naturally occurring disease in the United States and other regions worldwide and is recognized as a potential bioterrorism weapon. A bioweapon attack with *Y. pestis* could potentially infect thousands, requiring rapid and informed decision making by clinicians and public health agencies. The U.S. government stockpiles a variety of medical countermeasures to mitigate the effects of a bioterrorism attack (e.g., antimicrobials, antitoxins, and vaccines) for which the 21st Century Cures Act mandates the development of evidence-based guidelines on appropriate use. Guidelines for treatment and postexposure prophylaxis of plague were published in 2000 by a nongovernmental work group; since then, new human clinical data, animal study data, and U.S. Food and Drug Administration approvals of additional countermeasures have become available. To develop a comprehensive set of updated guidelines, CDC conducted a series of systematic literature reviews on human treatment of plague and other relevant topics to collect a broad evidence base for the recommendations in this report. Evidence from CDC reviews and additional sources were presented to subject matter experts during a series of forums. CDC considered individual expert input while developing these guidelines, which provide recommended best practices for treatment and prophylaxis of human plague for both naturally occurring disease and following a bioterrorism attack. The guidelines do not include information on diagnostic testing, triage decisions, or logistics involved in dispensing medical countermeasures. Clinicians and public health officials can use these guidelines to prepare their organizations, hospitals, and communities to respond to a plague mass-casualty event and as a guide for treating patients affected by plague.

## Introduction

### Plague Pathogenesis and Clinical Manifestations

*Yersinia pestis,* the causative agent of plague, is a nonmotile, gram-negative coccobacillus that persists in the natural environment in sylvatic cycles. Sporadic epizootics can sicken large numbers of rodents and other mammals and spill over to incidental hosts, including humans (*1*). *Y. pestis* can be transmitted to humans through the bite of an infected vector, namely the Oriental rat flea (*Xenopsylla cheopis*) and other flea species. Humans also can be infected via direct contact with infected tissues or fluids or inhalation of infectious droplets. In infected persons, the primary clinical form of plague depends on the route of transmission (*1*,*2*). Primary clinical presentations of plague include bubonic, pneumonic, septicemic (fever and sepsis without localizing signs), meningeal, and pharyngeal (pharyngitis with or without cervical lymphadenopathy) (*2*).

The most common clinical presentation of plague in humans is bubonic plague (*2*,*3*). Following the bite of an infected flea or direct contamination of a skin lesion, bacteria enter the host and are phagocytosed by macrophage and neutrophil cells (*4*). Surviving in macrophage cells, *Y. pestis* is then transported via lymphatics to regional lymph nodes (*4*,*5*). Numerous antiphagocytic factors protect *Y. pestis* from human immune responses and allow reproduction and spread of the bacteria (*6*). Within the lymph node, the bacteria multiply, producing a tender swelling or “bubo” (*5*). Persons with bubonic plague might experience additional symptoms of fever, chills, malaise, and headache 2–8 days after initial infection (*7*). If not treated promptly, rapid multiplication of bacteria in the lymph nodes causes destruction of the lymph node architecture and necrosis. Hematogenous spread of bacteria might lead to secondary septicemic, pneumonic, or meningeal plague. Left untreated, the fatality rate of bubonic plague is 66%; however, with antimicrobial treatment, the fatality rate decreases substantially to 13% (*3*).

Primary pneumonic plague develops following inhalation of *Y. pestis* from an infected animal or human. Within 1–3 days after inhalation, the infected person experiences fever, dyspnea, and, in late stages, a productive cough with purulent or bloody sputum (*6*,*8*,*9*). Unless antimicrobial treatment is initiated promptly, patients with pneumonic plague progress to respiratory failure, sepsis, and rapid death (*9*–*11*). Untreated pneumonic plague is almost always fatal (*9*,*10*).

Primary septicemic plague is characterized by *Y. pestis* infection of the bloodstream without appreciable lymphadenopathy, pneumonia, or other localizing signs. Patients often present with fever and gastrointestinal symptoms such as abdominal pain, nausea, vomiting, or diarrhea (*1**,**2*). Septicemic plague is frequently associated with delays in diagnosis and has a higher fatality rate than primary bubonic plague (*1**–**3*). 

Less common clinical presentations of plague include meningitis and pharyngitis. Plague meningitis typically occurs as a complication of delayed or inadequate treatment of another clinical form of primary plague and is characterized by fever, nuchal rigidity, and confusion (*2*). Cerebrospinal fluid neutrophilic pleocytosis combined with *Y. pestis* bacteria visible by Gram stain is a hallmark of plague meningitis. In one case series of 105 U.S. patients with plague, 6% developed meningitis, which most commonly appeared 9–14 days after the onset of acute *Y. pestis* infection among patients aged 10–15 years (*12*). Plague pharyngitis occurs following contamination of the oropharynx with *Y. pestis*-infected materials such as inadequately cooked meat of infected animals; typical symptoms include pharyngeal inflammation and cervical lymphadenopathy (*2*).

Before the advent of antibiotics, treatment of plague primarily consisted of supportive care, and fatality rates were high, between 66% and 93%, for all clinical forms (*3*,*13*). The development of sulfonamide antibiotics in the 1930s revolutionized the treatment of plague. Patients with pneumonic plague, previously considered incurable, were able to recover when treated within the first 24 hours of symptom onset (*14*,*15*). In the subsequent decades, the availability of aminoglycosides, followed by chloramphenicol, tetracyclines, and fluoroquinolones, offered additional options for plague treatment (*16*–*18*).

Naturally occurring plague frequently affects children. Among 658 patients with plague and recorded age identified in a systematic literature review, 265 (40%) were aged <18 years (*19*). However, children with plague do not appear to be at greater risk for death or serious complications compared with adults (*16*,*19*), although some evidence indicates children might be more likely to develop secondary plague meningitis (*12*,*20*).

Pregnant women with plague have experienced hemorrhage (i.e., postpartum hemorrhage and hemorrhage into tissues discovered on autopsy), intrauterine fetal demise, preterm birth, and stillbirth. Maternal-fetal transmission of *Y. pestis* has been reported for untreated mothers, although not when an infected mother received appropriate antimicrobial therapy (*21*). Information is limited on plague in other special populations, such as immunocompromised patients.

### Person-to-Person Transmission of Plague

Pneumonic plague is the only clinical form that is transmissible from person to person (*2*,*9*). *Y. pestis* is transmitted via large respiratory droplets that do not remain suspended in the air for prolonged periods. For this reason, health care providers should use respiratory droplet precautions when caring for patients with pneumonic plague (*8*). No evidence exists for airborne transmission of *Y. pestis*, as occurs with the measles virus or other pathogens that require airborne precautions to prevent exposure to others from an infected patient (*2*). Person-to-person transmission of *Y. pestis* requires exposure within 6 feet (*2*) and has been reported most commonly among an infected patient’s caregivers or others living together (*10*,*22*). 

A review of surveillance data during 1900–2009 yielded an estimated basic reproductive number (R_0_, the average number of secondary cases per case) of 1.18 for pneumonic plague in the United States, indicating that a patient with pneumonic plague will on average infect approximately one additional person (*23*). However, the average value obscures the high variability in the transmission potential of individual patients. The majority of U.S. patients do not transmit disease; however, this is offset by occasional superspreading events (*9*,*23*). All recorded superspreading events in the United States occurred before 1925, and it is likely that poor ventilation in residences and hospitals contributed to the propagation of these events (*9*). R_0_ can be reduced to <1 by implementing standard control measures, such as avoiding proximity to a coughing patient and instructing both the patient and those in their proximity to wear a surgical mask or cloth mask made of tightly woven, multilayered, breathable fabric (*2*,*9*,*23*).

During the initial 24 hours after a person has inhaled *Y. pestis*, risk for transmission is minimal because bacterial load in the lungs is low and cough has not yet fully developed (*9*). Asymptomatic person-to-person transmission of pneumonic plague has not been documented (*5*,*9*). Transmission risk increases as pneumonic plague progresses and is highest during mid-to-late stages of infection when patients cough sputum containing large amounts of bacteria (*2*,*24*). During terminal stages of infection, when patients are near death, the risk for transmission is likely reduced because patients can no longer cough vigorously and expel infectious droplets (*2*,*24*).

Person-to-person transmission of plague has occurred after patients with unrecognized pneumonic plague came into close (<6 feet) contact with family members, medical providers, or others. In 2014, the first possible case of person-to-person transmission in the United States since 1924 was documented in a woman who had extended close contact with an infected man while he was coughing bloody sputum. She recovered after treatment with levofloxacin, streptomycin, and doxycycline (*25*). Person-to-person transmission of pneumonic plague also has been documented in Madagascar (*10*,*26*), Peru (*27*), China (*28*,*29*), and Uganda (*22*).

### *Yersinia pestis* as a Biologic Weapon

*Y. pestis* is categorized as a Tier 1 bioterrorism Select Agent, the highest risk category of biologic agents and toxins with the potential to pose a severe threat to public health and safety (*30*). This designation is due in part to its low infectious dose, high case-fatality rate in untreated infection, and history of use as an agent of bioterrorism (*31*,*32*). The most concerning scenario for a bioterrorist attack involves dispersing *Y. pestis* into the air, leading to primary pneumonic plague among exposed persons. The World Health Organization (WHO) has estimated that a release of 50 kg of *Y. pestis* into the air over a city of 5 million persons could result in 150,000 cases of pneumonic plague and 36,000 deaths (*33*,*34*). Moreover, infection of animals with *Y. pestis* after such an attack could spark a local epizootic, resulting in primary bubonic plague among persons who handle infected animal carcasses or are bitten by infected fleas. Intentional contamination of the food or water supply with *Y. pestis* also has been raised as a potential concern and could lead to primary pharyngeal, bubonic, or septicemic plague.

Efforts to use *Y. pestis* as a biologic weapon have occurred for centuries. As early as 1346 CE, the Tartar army succeeded in conquering the Genoese port city of Caffa (present-day Theodosia, Ukraine) after hurling plague-infected corpses over the city’s walls, thereby initiating an outbreak of plague among its inhabitants and causing their subsequent retreat (*35*). During World War II, a covert branch of the Japanese army, known as Unit 731, conducted several aerial strikes by dropping millions of plague-infected fleas throughout China as part of its biologic warfare research program. Thousands of people became ill as a result of the ensuing outbreaks of plague, establishing these events as some of the largest and deadliest uses of *Y. pestis* as a bioweapon (*35*,*36*).

In the 1970s and 1980s, the Union of Soviet Socialist Republics manufactured large quantities of antibiotic-resistant *Y. pestis* for dissemination into the air as a bioweapon (*37*). In light of these and recent events involving terrorist organizations (*38*,*39*), the United States and other countries must be prepared for a bioterrorism attack involving *Y. pestis*.

### Rationale for Guideline Development

The U.S. Government has committed substantial resources to research, develop, procure, and stockpile medical countermeasures (MCMs) to mitigate the effects of possible chemical, biologic, radiologic, and nuclear threats to the public. These MCMs are maintained in the Strategic National Stockpile (SNS) for use during public health emergencies and can also be deployed for individual cases or small outbreaks if necessary (*40*). Under the 21st Century Cures Act, the U.S. Department of Health and Human Services, specifically CDC, develops and maintains timely and accurate guidelines for use of MCMs to treat and prevent diseases caused by bioterrorism agents.*

Existing guidelines for antimicrobial treatment and postexposure prophylaxis of plague in the United States are based largely on recommendations published in 2000 by the Working Group on Civilian Biodefense (*8*). Those recommendations incorporated input from CDC plague subject matter experts and others in the fields of clinical medicine, bioterrorism preparedness, and public health. However, since that publication, there have been many developments in plague treatment and prophylaxis. Additional human clinical data and animal study data have become available, and the Food and Drug Administration (FDA) approved several fluoroquinolones for prophylaxis and treatment of plague during 2012–2015 (*41*–*43*). Moreover, additional safety data about serious adverse effects of various antimicrobials used to treat plague have become available (*44*). In light of these developments, CDC re-examined the evidence on treatment and prophylaxis of plague and developed updated guidelines through a systematic process.

### Scope and Audience

These guidelines are intended to provide U.S. clinicians, public health practitioners, and first responders with evidence-based recommendations for the treatment and prophylaxis of plague in the event of an intentional release of *Y. pestis*. This report also outlines recommendations for management of naturally occurring plague. The guidelines could be adapted for use in other countries, with appropriate considerations for local health care practices and antimicrobial availability. These recommendations are not intended as a substitute for professional medical judgment or risk-benefit analysis for individual patients. Although these guidelines incorporate considerations for emergency situations, they do not include detailed recommendations for crisis standards of care, defined as a substantial change in usual health care operations and the level of care possible to deliver, made necessary by a pervasive or catastrophic (e.g., pandemic influenza or hurricane) disaster (*45*).

## Methods

CDC developed these guidelines after reviewing existing data on treatment and prophylaxis of plague, collecting and summarizing additional evidence, and gathering input from approximately 90 experts in numerous fields, including infectious diseases, emergency medicine, pharmacology, neonatology, obstetrics and gynecology, geriatrics, microbiology, epidemiology, and crisis standards of care. A CDC steering committee, comprising 10 experienced CDC and Office of the Assistant Secretary for Preparedness and Response (ASPR) staff members with diverse expertise, provided oversight and direction throughout the clinical guideline development process. The guideline development process included three major components: 1) systematic literature reviews, 2) two topic sessions, and 3) one expert forum.

To ensure these guidelines were based on the best available evidence, CDC’s Plague Clinical Guidelines Team conducted a systematic literature review of published cases of human plague with associated antimicrobial treatment information. Individual-level data from published case reports, case series, cohort studies, and randomized controlled trials were included. The resulting published review described 762 patients with plague who received antimicrobial treatment (*19*). A separate review of aggregate-level data on treatment of plague also was compiled and published (*46*).

U.S. surveillance data also informed development of these guidelines. Plague is a nationally notifiable condition in the United States (*47*), and most case reports submitted to CDC include information on antimicrobial treatment and outcome of patients. To contribute to the evidence base on treatment of plague, epidemiologists from CDC’s Division of Vector-Borne Diseases analyzed treatment and outcomes among 533 reported U.S. cases of plague (*16*). To inform specific recommendations for pregnant women, the Plague Clinical Guidelines Team and other contributors conducted systematic literature reviews of plague among pregnant women (*21*) and of existing published safety data regarding antimicrobials suggested for the treatment and prophylaxis of plague among pregnant women (*48*).

In August 2018, CDC convened two topic sessions consisting of approximately 40 key subject matter experts and agency representatives each. The first session focused on the treatment and prophylaxis of plague among adults and children, and the second focused on neonates, breastfeeding infants, and pregnant or lactating women. During each session, attendees reviewed summarized data and provided individual input on clinical considerations and recommendations for treatment and prophylaxis. CDC developed draft treatment and prophylaxis recommendations incorporating input from these sessions and under the direction of the CDC steering committee, with the goal of further review and refinement of the recommendations during an expert forum with clinical and public health experts.

In May 2019, CDC convened an expert forum consisting of approximately 90 clinical and public health subject matter experts and agency representatives. Federal representatives from CDC, FDA, ASPR (including the Biomedical Advanced Research and Development Authority), the National Institutes of Health (NIH), and the U.S. Department of Defense were in attendance. Clinical organizations represented included the American Academy of Pediatrics, American College of Emergency Physicians, American College of Obstetricians and Gynecologists (ACOG), American Geriatrics Society, Infectious Diseases Society of America, Society of Critical Care Medicine, and the Society for Maternal-Fetal Medicine. Public health organizations represented included WHO, Pan American Health Organization, Council of State and Territorial Epidemiologists, Association of State and Territorial Health Officials, and the National Association of County and City Health Officials. Experts on plague and emergency preparedness from domestic and international universities, clinics, and hospitals also were in attendance to provide their expertise and opinions.

During the expert forum, subject matter experts from CDC, other federal agencies, and clinical organizations presented data from systematic literature reviews and U.S. surveillance reports. Information on antimicrobial risks and adverse effects also was reviewed and considered. Breakout sessions were held to discuss specific topics and special populations in further detail. Individual expert opinions on treatment and prophylaxis options were recorded and reviewed; no attempt was made to achieve consensus because final decisions about recommendations were determined solely by CDC.

Published data and gray literature on U.S. studies of antimicrobial treatment of plague in nonhuman primates also were considered during topic session and expert forum discussions. This included animal efficacy data from NIH that supported FDA’s approval of plague treatment and prophylaxis indications for ciprofloxacin, levofloxacin, and moxifloxacin. Evidence from these studies is summarized elsewhere (*49*). The Plague Clinical Guidelines Team also reviewed existing guidelines from WHO, the European Centre for Disease Prevention and Control, and select countries where plague is endemic.

All nonfederal staff, subject matter experts, and representatives from clinical organizations who participated in guideline development activities completed a questionnaire to review and mitigate potential competing interests. All federal employees, including CDC staff, are subject to the Standards of Ethical Conduct for Employees of the Executive Branch.^†^

Although all antimicrobials listed in these recommendations are FDA approved, some might not be readily available in the United States because of limited production. These antimicrobials were included to provide multiple options for treatment and prophylaxis of plague, especially in the event of a large-scale intentional release of *Y. pestis.* In this case, domestic supply of specific antimicrobials might need to be augmented through manufacturing surge and importation. These antimicrobials also can be useful in the setting of non-U.S. plague outbreaks.

## Recommendations

### Notable Changes and Updates

Since the publication of plague guidelines in 2000, FDA has approved ciprofloxacin, levofloxacin, and moxifloxacin for treatment and prophylaxis of plague in humans on the basis of the Animal Rule (*49*). FDA can rely on adequate and well-controlled animal efficacy studies to support approval of a drug or licensure of a biologic product under the Animal Rule when human efficacy studies are not ethical and field trials are not feasible (*50*,*51*).

Several clinical studies on treatment of plague in humans also have contributed to advancements in the field. A randomized controlled trial published in 2006 compared gentamicin with doxycycline for treatment of plague in Tanzania among 65 enrolled adults and children (*17*). Recent case series also have reported on the use of ciprofloxacin for treatment of bubonic and pneumonic plague in Uganda (*18*) and compared streptomycin with gentamicin for treatment of human plague in New Mexico (*52*).

On the basis of new developments, expert forum discussions, and other considerations, CDC made the following overarching changes compared with the guidelines published in 2000 by the Working Group on Civilian Biodefense (*8*):

1. Simplified the guidelines to treatment (intravenous [IV], intramuscular [IM], or oral options) and prophylaxis scenarios, similar to clinical guidelines for other biologic threats. Previously, guidelines were divided into contained casualty (all IV or IM treatment) versus mass-casualty (all oral treatment) scenarios (*8*). The rationale behind this shift is that it might not be clear initially how large the outbreak will be (i.e., whether it will remain a contained casualty event or evolve to a mass-casualty event). Because of this uncertainty, it might be advisable at the outset to treat patients with mild-to-moderate illness with oral therapy to conserve IV medications and supplies and avoid the risks for IV access to the patient, such as infection and bleeding. This new approach provides greater flexibility for responding to uncertain situations.
2. Added recommendations for treatment and prophylaxis of clinical forms of plague other than pneumonic, including bubonic, septicemic, pharyngeal, and meningeal. The recommendations were expanded because both intentional release of *Y. pestis* and naturally occurring infections can lead to various clinical manifestations.
3. Added recommendations for neonates and breastfeeding infants. The recommendations were expanded to add these populations because they might be impacted by naturally occurring infections or a bioterrorism attack and require different considerations for treatment and prophylaxis.
4. Listed ciprofloxacin as a first-line (preferred) agent for treatment of plague rather than an alternative option because more data are now available to support its use. Levofloxacin and moxifloxacin, which were not included in previous guidelines, are listed as first-line agents or alternatives for both treatment and prophylaxis.
5. Added several same-class alternatives to first-line fluoroquinolones, aminoglycosides, and tetracyclines to expand the repertoire of treatment and prophylaxis options to meet surge capacity, if needed. Although direct evidence of efficacy in humans or animals is lacking for some of these antimicrobials, those with similar mechanisms of action to first-line agents would be expected to be effective for plague and are supported by in vitro data.
6. Added the sulfonamide drug trimethoprim-sulfamethoxazole as an alternative option for prophylaxis of plague because of evidence of efficacy in humans (*19*,*46*) and its use as prophylaxis during naturally occurring plague outbreaks internationally (*26*,*53*).
7. Added recommendations for pre-exposure prophylaxis in addition to revising postexposure prophylaxis recommendations.

### Response to *Yersinia pestis* Release as a Biologic Weapon

A bioterrorism attack with *Y. pestis* could have a devastating impact on U.S. society. Intentional release of *Y. pestis* could occur via air, food, or even the release of infected fleas, with resultant clinical presentations of affected persons depending on route of exposure (*34*). Children might be at greater risk for infection after a bioterrorism attack compared with adults because of increased respiratory rate, larger relative body surface area, closer proximity to the ground and other surfaces, and additional factors (*54*).

A covert release of *Y. pestis* into the air would cause illness in infected persons within 1–3 days (*6*,*34*). Initial manifestations would be nonspecific (i.e., fatigue, fever, cough, and dyspnea) and mimic many respiratory illnesses. Chest radiographs of affected patients would reveal nonspecific disease processes such as nodular or patchy infiltrates, lobar pneumonia, pleural effusion, or hilar lymphadenopathy (*9*). Recognition of a bioterrorist attack would occur after an abnormal pattern of disease is detected and results of agent-specific diagnostic tests (e.g., polymerase chain reaction) become available.

Following a plague-related bioterrorism event, health care providers should treat symptomatic patients with two distinct classes of antimicrobials, at least one of which is considered first-line (Tables 1 and 2), until sensitivity patterns of the infecting *Y. pestis* strain are known. Although naturally occurring antimicrobial resistance is extremely rare in *Y. pestis* (*55*,*56*), there is potential for engineered resistance as part of an intentional bioterrorism release (*8*,*57*–*59*). Treating initially with two distinct classes of antimicrobials increases the likelihood that the patient will receive at least one effective agent.

**TABLE 1:** Treatment of adults and children with pneumonic or septicemic plague

Population	Category	Antimicrobial*^,†^ *Class*	Dosage^§^
**Adults** **aged ≥18 yrs**	**First-line**	Ciprofloxacin *Fluoroquinolone*	400 mg every 8 hrs IV or 750 mg every 12 hrs PO
		Levofloxacin *Fluoroquinolone*	750 mg every 24 hrs IV or PO
		Moxifloxacin *Fluoroquinolone*	400 mg every 24 hrs IV or PO
		Gentamicin^¶^ *Aminoglycoside*	5 mg/kg every 24 hrs IV or IM
		Streptomycin** *Aminoglycoside*	1 g every 12 hrs IV^††^ or IM
	**Alternatives**	Doxycycline *Tetracycline*	200 mg loading dose, then 100 mg every 12 hrs IV or PO
		Chloramphenicol^¶,^** *Amphenicol*	12.5–25 mg/kg every 6 hrs IV^§§^ (maximum 1 g/dose)
		Ofloxacin^¶,¶¶^ *Fluoroquinolone*	400 mg every 12 hrs PO***
		Gemifloxacin^¶^ *Fluoroquinolone*	320 mg every 24 hrs PO
		Amikacin^¶^ *Aminoglycoside*	15–20 mg/kg every 24 hrs IV or IM
		Tobramycin^¶^ *Aminoglycoside *	5–7 mg/kg every 24 hrs IV or IM
		Plazomicin^¶,^** *Aminoglycoside*	15 mg/kg every 24 hrs IV
		Trimethoprim-sulfamethoxazole^¶^ *Sulfonamide*	5 mg/kg (trimethoprim component) every 8 hrs IV or PO
**Children** ** aged ≥1 mos to ≤17 yrs (unless otherwise noted)**	**First-line**	Ciprofloxacin *Fluoroquinolone*^†††^	10 mg/kg every 8 or 12 hrs IV or 15 mg/kg every 8 or 12 hrs PO (maximum 400 mg/dose IV, 500 mg/dose every 8 hrs PO, or 750 mg/dose every 12 hrs PO)
		Levofloxacin *Fluoroquinolone*^†††^	Body weight <50 kg: 8 mg/kg every 12 hrs IV or PO (maximum 250 mg/dose) Body weight ≥50 kg: 500–750 mg every 24 hrs IV or PO
		Gentamicin^¶^ *Aminoglycoside*	4.5–7.5 mg/kg every 24 hrs IV or IM
		Streptomycin** *Aminoglycoside*	15 mg/kg every 12 hrs IV^††^ or IM (maximum 1 g/dose)
	**Alternatives**	Doxycycline *Tetracycline*^†††^	Body weight <45 kg: 4.4 mg/kg loading dose, then 2.2 mg/kg every 12 hrs IV or PO Body weight ≥45 kg: 200 mg loading dose, then 100 mg every 12 hrs IV or PO
		Chloramphenicol^¶,^** *Amphenicol*	12.5–25 mg/kg every 6 hrs IV^§§^ (maximum 1 g/dose)
		Moxifloxacin^§§§^ *Fluoroquinolone*^†††^	Infants and children aged ≥3 mos to ≤23 mos: 6 mg/kg every 12 hrs IV or PO^¶¶¶^ Children aged 2–5 yrs: 5 mg/kg every 12 hrs IV or PO^¶¶¶^ Children aged 6–11 yrs: 4 mg/kg every 12 hrs IV or PO^¶¶¶^ Children and adolescents aged 12 to ≤17 yrs: Body weight <45 kg: 4 mg/kg every 12 hrs IV or PO^¶¶¶^ Maximum dose for all children <45 kg: 200 mg/dose Body weight ≥45 kg: 400 mg every 24 hrs IV or PO^¶¶¶^
		Ofloxacin^¶,¶¶^ *Fluoroquinolone*^†††^	7.5 mg/kg every 12 hrs PO*** (maximum 400 mg/dose)
		Amikacin^¶^ *Aminoglycoside *	15–20 mg/kg every 24 hrs IV or IM
		Tobramycin^¶^ *Aminoglycoside*	4.5–7.5 mg/kg every 24 hrs IV or IM
		Trimethoprim-sulfamethoxazole^¶^ *Sulfonamide*	Infants and children aged ≥2 mos to ≤17 yrs: 5 mg/kg (trimethoprim component) every 8 hrs IV or PO

**Abbreviations:** IM = intramuscular; IV = intravenous; PO = per os.

**Note:** All oral antimicrobials recommended in these guidelines can be administered via alternative enteral routes (e.g., nasogastric tube and gastric tube) except for ciprofloxacin.

* Dual therapy with two distinct classes of antimicrobials should be used for initial treatment of patients with severe pneumonic or septicemic plague and patients infected after intentional release of *Yersinia pestis*.

^†^ Antimicrobials are not listed in order of preference within each category.

^§^ Recommended treatment duration is 10–14 days.

^¶^ Not approved by the Food and Drug Administration (FDA) for treatment of plague. In some instances, these antimicrobials have been used off label for the treatment of naturally occurring plague (*17**,**19**,**88*). Large-scale distribution and use of these antimicrobials during a bioterrorism response might be under FDA-issued Emergency Use Authorization.

** At the time of publication, these antimicrobials might not be readily and consistently available in the United States because of limited production.

^††^ The IV formulation of streptomycin is not approved by FDA; however, the IM formulation of streptomycin has been given intravenously as an off-label use (**Sources:** Morris JT, Cooper RH. Intravenous streptomycin: a useful route of administration. Clin Infect Dis 1994;19:1150–1. Pérez Tanoira R, Sánchez-Patán F, Jiménez Girón A. et al. Tolerance and safety of intravenous streptomycin therapy in patients with tuberculosis. Infection 2014;42:597–8).

^§§^ The lower end of the chloramphenicol dosing range (12.5 mg/kg every 6 hours) is sufficient for treatment of plague in most cases. Severe infections might require increased dosing, but these doses should be decreased as soon as feasible (**Sources:** Chloramphenicol sodium succinate [Package insert]. Lake Zurich, IL: Fresenius Kabl, LLC; 2019. Chloromycetin sodium succinate [Package insert]. Bristol, TN: Monarch Pharmaceuticals, LLC; 2004). Serum concentration monitoring should be performed when available, especially in children.

^¶¶^ Additional fluoroquinolone alternatives, such as delafloxacin, also can be considered depending on drug availability.

*** Ofloxacin suspension for oral liquid administration is not available in the United States.

^†††^ Data on use of fluoroquinolones and doxycycline in infants and young children are limited.

^§§§^ Moxifloxacin is not FDA approved for use in children aged ≤17 years but has been used off label (*79*). Data on use in neonates and children aged ≤2 months are extremely limited; however, successful use in neonates has been reported (**Source:** Watt KM, Massaro MM, Smith B, Cohen-Wolkowiez M, Benjamin DK Jr, Laughon MM. Pharmacokinetics of moxifloxacin in an infant with *Mycoplasma hominis* meningitis. Pediatr Infect Dis J 2012;31:197–9). For children aged 12–17 years weighing ≥45 kg with risk factors for cardiac events, consider 200 mg twice daily to reduce risk for QT prolongation.

^¶¶¶^ Although no commercial liquid formulation is available for moxifloxacin, hospitals and compounding retail pharmacies can use a published recipe to make liquid suspension.

**TABLE 2:** Treatment of adults and children with bubonic or pharyngeal plague

Population	Category	Antimicrobial*^,†^ *Class*	Dosage^§^
**Adults aged ≥18 yrs**	**First-line**	Ciprofloxacin *Fluoroquinolone*	400 mg every 8 hrs IV or 750 mg every 12 hrs PO
		Levofloxacin *Fluoroquinolone*	750 mg every 24 hrs IV or PO
		Moxifloxacin *Fluoroquinolone*	400 mg every 24 hrs IV or PO
		Doxycycline *Tetracycline*	200 mg loading dose, then 100 mg every 12 hrs IV or PO
		Gentamicin^¶^ *Aminoglycoside*	5 mg/kg every 24 hrs IV or IM
		Streptomycin** Aminoglycoside	1 g every 12 hrs IV^††^ or IM
	**Alternatives**	Chloramphenicol^¶,^** *Amphenicol*	12.5–25 mg/kg every 6 hrs IV^§§^ (maximum 1 g/dose)
		Ofloxacin^¶,¶¶^ *Fluoroquinolone*	400 mg every 12 hrs PO***
		Gemifloxacin^¶^ *Fluoroquinolone*	320 mg every 24 hrs PO
		Amikacin^¶^ *Aminoglycoside*	15–20 mg/kg every 24 hrs IV or IM
		Tobramycin^¶^ *Aminoglycoside*	5–7 mg/kg every 24 hrs IV or IM
		Plazomicin^¶,^** Aminoglycoside	15 mg/kg every 24 hrs IV
		Tetracycline^¶^ *Tetracycline*	500 mg every 6 hrs PO
		Omadacycline^¶^ *Tetracycline*	200 mg loading dose on day 1, then 100 mg every 24 hrs IV or 450 mg loading dose every 24 hrs on days 1 and 2, then 300 mg every 24 hrs PO
		Minocycline^¶^ *Tetracycline*	200 mg loading dose, then 100 mg every 12 hrs IV or PO
		Eravacycline^¶^ *Tetracycline *	1 mg/kg every 12 hrs IV
		Trimethoprim-sulfamethoxazole^¶^ *Sulfonamide*	5 mg/kg (trimethoprim component) every 8 hrs IV or PO
**Children aged ≥**1 **mos to ≤17 yrs (unless otherwise noted)**	**First-line**	Ciprofloxacin *Fluoroquinolone*^†††^	10 mg/kg every 8 or 12 hrs IV 15 mg/kg every 8 or 12 hrs PO (maximum 400 mg/dose IV, 500 mg/dose every 8 hrs PO or 750 mg/dose every 12 hrs PO)
		Levofloxacin *Fluoroquinolone*^†††^	Body weight <50 kg: 8 mg/kg every 12 hrs IV or PO (maximum 250 mg/dose) Body weight 50 kg: 500–750 mg every 24 hrs IV or PO
		Doxycycline *Tetracycline*^†††^	Body weight <45 kg: 4.4 mg/kg loading dose, then 2.2 mg/kg every 12 hrs IV or PO Body weight ≥45 kg: 200 mg loading dose, then 100 mg every 12 hrs IV or PO
		Gentamicin^¶^ *Aminoglycoside*	4.5–7.5 mg/kg every 24 hrs IV or IM
		Streptomycin** Aminoglycoside	15 mg/kg every 12 hrs IV^††^ or IM (maximum 1 g/dose)
	**Alternatives**	Chloramphenicol^¶,^** *Amphenicol*	12.5–25 mg/kg every 6 hrs IV^§§^ (maximum 1 g/dose)
		Moxifloxacin^§§§^ *Fluoroquinolone*^†††^	Infants and children aged ≥3 mos to ≤23 mos: 6 mg/kg every 12 hrs IV or PO^¶¶¶^ Children aged 2–5 yrs: 5 mg/kg every 12 hrs IV or PO^¶¶¶^ Children aged 6–11 yrs: 4 mg/kg every 12 hrs IV or PO^¶¶¶^ Children and adolescents aged 12 to ≤17 yrs: Body weight <45 kg: 4 mg/kg every 12 hrs IV or PO^¶¶¶^ Maximum dose for all children <45 kg: 200 mg/dose Body weight ≥45 kg: 400 mg every 24 hrs IV or PO^¶¶¶^
		Ofloxacin^¶,¶¶^ *Fluoroquinolone*^†††^	7.5 mg/kg every 12 hrs PO*** (maximum 400 mg/dose)
		Amikacin^¶^ *Aminoglycoside*	15–20 mg/kg every 24 hrs IV or IM
		Tobramycin^¶^ *Aminoglycoside*	4.5–7.5 mg/kg every 24 hrs IV or IM
		Tetracycline^¶^ *Tetracycline*^†††^	10 mg/kg every 6 hrs PO (maximum 500 mg/dose)
		Minocycline^¶^ *Tetracycline*^†††^	4 mg/kg loading dose (maximum dose 200 mg), then 2 mg/kg every 12 hrs IV or PO (maximum 100 mg/dose)
		Trimethoprim-sulfamethoxazole^¶^ *Sulfonamide*	Infants and children aged ≥2 mos to ≤17 yrs: 5 mg/kg (trimethoprim component) every 8 hrs IV or PO

**Abbreviations:** IM = intramuscular; IV = intravenous; PO = per os.

**Note:** All oral antimicrobials recommended in these guidelines can be administered via alternative enteral routes (e.g., nasogastric tube and gastric tube) except for ciprofloxacin.

*Monotherapy is recommended for patients with naturally occurring plague, although dual therapy can be considered for patients with large buboes. Dual therapy with two distinct classes of antimicrobials should be used for initial treatment of patients infected after intentional release of *Yersinia pestis*.

^†^ Antimicrobials are not listed in order of preference within each category.

^§^ Recommended treatment duration is 10–14 days.

^¶^ Not approved by the Food and Drug Administration (FDA) for treatment of plague. In some instances, these antimicrobials have been used off label for the treatment of naturally occurring plague (*17**,**19**,**88*). Large-scale distribution and use of these antimicrobials during a bioterrorism response might be under FDA-issued Emergency Use Authorization.

** At the time of publication, these antimicrobials might not be readily and consistently available in the United States because of limited production.

^††^ The IV formulation of streptomycin is not approved by FDA; however, the IM formulation of streptomycin has been given intravenously as an off-label use (**Sources:** Morris JT, Cooper RH. Intravenous streptomycin: a useful route of administration. Clin Infect Dis 1994;19:1150–1. Pérez Tanoira R, Sánchez-Patán F, Jiménez Girón A. et al. Tolerance and safety of intravenous streptomycin therapy in patients with tuberculosis. Infection 2014;42:597–8).

^§§^ The lower end of the chloramphenicol dosing range (12.5 mg/kg every 6 hours) is sufficient for treatment of plague in most cases. Severe infections might require increased dosing, but these doses should be decreased as soon as feasible. (**Sources:** Chloramphenicol sodium succinate [Package insert]. Lake Zurich, IL: Fresenius Kabl, LLC; 2019. Chloromycetin sodium succinate [Package insert]. Bristol, TN: Monarch Pharmaceuticals, LLC; 2004). Serum concentration monitoring should be performed when available, especially in children.

^¶¶^ Additional fluoroquinolone alternatives, such as delafloxacin, also can be considered depending on drug availability.

*** Ofloxacin suspension for oral liquid administration is not available in the United States.

^†††^ Data on use of fluoroquinolones and tetracyclines in infants and young children are limited. Because of the risk for permanent tooth discoloration and tooth enamel hypoplasia, tetracycline and minocycline should only be used for children aged <8 years when other treatment options have been exhausted.

^§§§^ Moxifloxacin is not FDA approved for use in children aged ≤17years but has been used off label (*79*). Data on use in neonates and children aged ≤2 months are extremely limited; however, successful use in neonates has been reported (**Source:** Watt KM, Massaro MM, Smith B, Cohen-Wolkowiez M, Benjamin DK Jr, Laughon MM. Pharmacokinetics of moxifloxacin in an infant with *Mycoplasma hominis* meningitis. Pediatr Infect Dis J 2012;31:197–9). For children aged 12–17 years weighing ≥45 kg with risk factors for cardiac events, consider 200 mg twice daily to reduce risk for QT prolongation.

^¶¶¶^ Although no commercial liquid formulation is available for moxifloxacin, hospitals and compounding retail pharmacies can use a published recipe to make liquid suspension.

Once information from antimicrobial susceptibility testing becomes available, this will be a factor in determining which antimicrobials should be continued or instituted. Antimicrobial susceptibility testing might not be required for all patients when initial susceptibility results are available, if the attack is determined to be a single release.

In the aftermath of an intentional release of *Y. pestis*, antimicrobial prophylaxis also should be considered for potentially exposed persons. Risk stratification and eligibility criteria for receipt of antimicrobial prophylaxis among exposed persons would be developed and determined by federal, state, and local public health agencies on the basis of the nature and extent of the incident, available resources, and an ethical framework similar to other countermeasures.

### Antimicrobial Treatment of Adults and Children

A variety of antimicrobial classes are effective for plague. FDA-approved antimicrobials for treatment and prophylaxis of plague include streptomycin, ciprofloxacin, levofloxacin, moxifloxacin, and doxycycline. Although gentamicin, chloramphenicol, and trimethoprim-sulfamethoxazole are not FDA approved for plague, they are considered to be effective on the basis of clinical experience and animal data.

Aminoglycosides in particular have a long history of successful use for all predominant forms of plague as well as robust published human clinical data (*19*,*46*,*60*,*61*) and animal data supporting efficacy (*49*,*62*). Among reported U.S. cases of plague, 61 (84%) of 73 patients survived when treated with aminoglycoside monotherapy, defined as receiving no additional antimicrobial considered to be effective for treating plague. In addition, a retrospective case series comparing streptomycin with gentamicin among 50 patients with plague in New Mexico found that patients treated with gentamicin had similar fever duration, number of complications, and survival rates compared with those who received streptomycin (*52*). However, aminoglycosides might be less desirable than other antimicrobials in certain instances because of the lack of oral formulations and potential for adverse effects such as nephrotoxicity and ototoxicity (*63*,*64*). When treating patients with aminoglycosides, clinicians should check drug concentrations as indicated on the basis of dosing strategy (e.g., extended interval or traditional dosing) and adjust dose and dosing interval accordingly (*65*).

Tetracyclines, sulfonamides, and chloramphenicol also have been used to treat plague successfully for many years. A review of published cases of plague found that survival rates of patients treated with tetracycline, sulfonamide, or chloramphenicol monotherapy were 90% (n = 44 of 49), 70% (n = 151 of 216), and 75% (n = 15 of 20), respectively (*19*). Analysis of 533 U.S. cases of plague reported during 1942–2018 demonstrated survival rates of patients treated with tetracycline, sulfonamide, or chloramphenicol monotherapy were 98% (n = 50 of 51), 82% (n = 9 of 11), and 78% (n = 7 of 9), respectively. Multivariable adjustment controlling for primary clinical form of plague (bubonic versus nonbubonic), time, secondary plague manifestations, and illness complications found that tetracyclines were associated with the highest adjusted odds of survival (aOR: 6.2; 95% confidence interval [CI] = 2.6–15.1) among 469 patients receiving any antimicrobial treatment (*16*). However, absolute survival rates of patients treated with sulfonamides or chloramphenicol might be artificially decreased because of time bias, since these antimicrobials were primarily used before 1980.

In recent years, investigators have performed additional studies to evaluate the efficacy of fluoroquinolones for pneumonic plague in nonhuman primates with promising results (*49*,*66*,*67*). Moreover, a case series published in 2017 described five Ugandan patients with culture-confirmed plague who survived after oral ciprofloxacin treatment. One of these successfully treated patients had pneumonic plague with hemoptysis and bilateral infiltrates on chest radiograph (*18*).

Penetration of antimicrobials varies by tissue type and specific drug, and some human and animal data suggest that the relative efficacy of various antimicrobials differs by clinical form of plague (*16*,*19*). For these reasons, treatment recommendations are separated into those for pneumonic and septicemic, bubonic and pharyngeal, and meningeal plague. Recommendations for pneumonic and septicemic plague are combined because these are the most severe and rapidly progressive forms of disease, and patients with septicemic plague can have subclinical pneumonic infection (*68*). Recommendations for pharyngeal plague are combined with those for bubonic because it is a milder form of disease often accompanied by focal lymphadenopathy (*61*,*69*).

Clinicians should use their judgment and these guidelines to decide whether to initiate parenteral or oral antimicrobials to treat patients with plague, depending on the severity of disease and whether the patient can tolerate oral medications. Patients initially treated intravenously can be transitioned to the oral route, if deemed appropriate by the health care team, when clinical improvement is apparent. Except for ciprofloxacin, all oral antimicrobials recommended in these guidelines can be administered via alternative enteral routes (e.g., nasogastric tube and gastric tube). Treatment duration for all clinical forms of plague should be 10–14 days total; treatment duration can be extended for patients with ongoing fever or other concerning signs or symptoms.

### Pneumonic and Septicemic Plague

Among 158 published cases of primary pneumonic plague analyzed as part of a systematic literature review, patients who received aminoglycosides, fluoroquinolones, or tetracyclines, alone or in combination with other antimicrobials had the highest survival rates: 83% (n = 81 of 98), 82% (n = 28 of 34), and 82% (n = 23 of 28), respectively (*19*). Survival rates of patients who received chloramphenicol and sulfonamides, alone or in combination with other antimicrobials, were 78% (n = 25 of 32) and 65% (n = 28 of 43), respectively. When limited to patients who received monotherapy only, survival rates were 81% (n = 26 of 32) for aminoglycosides, 80% (n = 4 of 5) for fluoroquinolones, 83% (n = 5 of 6) for tetracyclines, 67% (n = 8 of 12) for chloramphenicol, and 50% (n = 13 of 26) for sulfonamides (*19*).

A total of 97 patients with primary pneumonic and septicemic plague were reported to CDC during 1942–2018 via U.S. surveillance (*16*). Among these, survival rates were highest for patients treated with sulfonamides (100%; n = 5 of 5), tetracyclines (93%; n = 39 of 42), and fluoroquinolones (91%; n = 21 of 23) alone or in combination with other antimicrobials. Survival rates for patients treated with aminoglycosides and chloramphenicol were 80% (n = 49 of 61) and 60% (n = 9 of 15), respectively. However, these observed differences in survival rates might be attributed in part to differences in when patients initially sought care and the severity of their illness at presentation. For example, patients with mild-to-moderate infections might have been preferentially treated with an oral antimicrobial such as doxycycline, whereas sicker patients might have been more likely to receive aminoglycosides (*16*).

Studies of nonhuman primates have raised questions about the efficacy of tetracyclines for treating pneumonic plague in humans (*49*). Three distinct studies of African green monkeys yielded poor outcomes; however, each was complicated by pharmacokinetic anomalies. In the first study, 44% (4 of 9) of African green monkeys treated with oral doxycycline 6–6.5 hours after fever onset survived. Measured drug serum levels were below those expected in humans (*49*). In two follow-up studies with adjusted dosing of oral or IV doxycycline, none of 10 monkeys survived. Toxicity due to drug accumulation in the animals could not be excluded (*49*). Switching to cynomolgus macaques, a study found that 88% (7 of 8) of macaques treated with IV doxycycline survived when treatment was initiated 6 hours after fever onset. Similarly, 75% (6 of 8) of macaques treated with IV doxycycline survived when treatment was initiated 15 hours after fever onset (*49*). On the basis of these animal model data and expert opinion, doxycycline is listed as an alternative option for treatment of pneumonic or septicemic plague.

Additional fluoroquinolones and aminoglycosides can serve as within-class alternatives to first-line agents for primary pneumonic and septicemic plague in humans. Plazomicin has been shown to be effective for treatment of pneumonic plague in African green monkeys (*70*). Tobramycin, amikacin, and ofloxacin are believed to be effective by extrapolation of data from first-line aminoglycosides and fluoroquinolones. However, these recommendations do not include an exhaustive list of all potential within-class alternatives. Additional existing or new antimicrobials within the same class of first-line agents could be considered along with expert consultation should the need arise.

Treatment recommendations for patients with septicemic or pneumonic plague, either naturally occurring or following intentional release of *Y. pestis*, are summarized (Table 1). If clinical and exposure history clearly indicate naturally occurring plague (e.g., close contact [<6 feet] with a severely ill and coughing patient with known naturally acquired pneumonic plague, or close or direct contact with an animal with plague), health care providers can consider treating patients with mild-to-moderate disease with a single recommended antimicrobial agent. For patients with severe septicemic or pneumonic disease, clinicians should institute dual therapy with two distinct antimicrobial classes, with narrowing of therapy to a single antimicrobial after clinical improvement.

When caring for patients with naturally occurring pneumonic plague, if diagnosis is uncertain, health care providers should include antimicrobial coverage for community-acquired pneumonia until results of diagnostic testing are available. Levofloxacin or moxifloxacin are particularly good options in these circumstances because they have robust lung penetration (*71*–*73*) and activity against gram-positive bacteria, gram-negative bacteria, and atypical pathogens that cause community-acquired pneumonia (*74*,*75*). 

Naturally occurring resistance of *Y. pestis* to antimicrobials typically used to treat plague is rare worldwide and has not been reported in the United States (*55*,*56*,*76*,*77*). However, engineered resistance in *Y. pestis* has been demonstrated and remains a potential threat (*8*,*37*,*59*). Therefore, these guidelines include recommendations to treat patients with two distinct classes of antimicrobials if there is reason to suspect antimicrobial resistance was engineered as part of a bioterrorism attack.

For children aged ≥3 months to 17 years, moxifloxacin is recommended as an alternative antimicrobial rather than a first-line agent because of lack of FDA approval for use in children and higher reported rates of prolonged QTc interval compared with other fluoroquinolones (*78*–*80*). Moreover, plazomicin is not recommended as an alternative antimicrobial for children aged ≥1 month to 17 years because no published data exists on its use and dosage in the pediatric population.

Because of concerns about tooth staining or enamel hypoplasia, clinicians have been hesitant to prescribe doxycycline to children aged <8 years (*81*). However, this tetracycline class warning was based on clinical experiences with older tetracyclines that bind calcium more readily than doxycycline. A recent study found no evidence of dental staining or enamel hypoplasia among children who received short-term courses (≤21 days) of doxycycline compared with those who never received doxycycline (*82*). For life-threatening infections such as plague, use of doxycycline in children aged <8 years is justified on the basis of a favorable risk-benefit ratio. Other tetracycline class drugs, such as tetracycline and minocycline, should only be used for children aged <8 years when other treatment or prophylaxis options have been exhausted.

### Bubonic and Pharyngeal Plague

In general, treatment for bubonic and pharyngeal plague, either naturally occurring or after an intentional release of *Y. pestis*, is similar to pneumonic and septicemic plague. Antimicrobials with demonstrated robust in vitro and in vivo activity against *Y. pestis* are preferred for treatment of bubonic and pharyngeal plague (Table 2). However, a notable difference in the recommendations for bubonic and pharyngeal disease is that doxycycline is considered a first-line treatment. Although certain studies have demonstrated reduced doxycycline efficacy for pneumonic plague (*49*,*67*), doxycycline remains a safe and effective treatment for primary bubonic plague among patients who have not progressed to severe secondary septicemic or pneumonic plague. In a randomized clinical trial in Tanzania comparing gentamicin with doxycycline, 29 (97%) of 30 patients with bubonic plague survived after oral doxycycline treatment. The one death in the doxycycline group was attributed to advanced disease in a woman with a recent spontaneous abortion, a retained dead fetus, hemorrhage, and renal failure at the time treatment was initiated (*17*). Moreover, an analysis of U.S. surveillance data demonstrated that, among a subset of 45 patients with bubonic plague treated with tetracyclines and no other antimicrobial classes effective for plague, 44 (98%) survived (*16*) (Supplementary Appendix 1, https://stacks.cdc.gov/view/cdc/107427). On the basis of this demonstrated clinical efficacy and in vitro activity against *Y. pestis* (*55*,*76*,*83*), doxycycline is recommended as a first-line antimicrobial for treatment of patients with primary bubonic or pharyngeal plague.

Abscesses such as buboes have lower pH than most human tissues (reported pH range: 5.5–7.2) (*84*). In acidic environments, transport of aminoglycosides into the bacterial cell is reduced, leading to suppressed activity of the drug (*85*,*86*). This has raised the question of whether aminoglycosides are effective for treatment of bubonic plague. However, clinical evidence on use of aminoglycosides for bubonic plague does not demonstrate marked reductions in efficacy. In the previously mentioned Tanzania trial, 33 (94%) of 35 patients with bubonic plague survived with gentamicin treatment (*17*). The two fatalities in the gentamicin group had advanced disease and died within 4 hours of the first dose of gentamicin (*17*). Among reported U.S. cases of primary bubonic plague, 50 (91%) of 55 patients survived when treated with aminoglycoside monotherapy (i.e., received no additional antimicrobial considered to be effective for treatment of plague). In comparison, eight (80%) of 10 patients treated with sulfonamide, 44 (98%) of 45 patients treated with tetracycline, and all four patients treated with fluoroquinolone monotherapy for bubonic plague survived, although treatment bias and other factors might have contributed to some differences in survival rates (Supplementary Appendix 1, https://stacks.cdc.gov/view/cdc/107427). In a systematic literature review of published cases of plague, 57 (83%) of 69 patients with primary bubonic plague survived when treated with aminoglycoside monotherapy. In comparison, survival of patients with primary bubonic plague treated with sulfonamide, tetracycline, or fluoroquinolone monotherapy was 75% (119 of 159), 95% (36 of 38), and 100% (14 of 14), respectively (*19*).

Because of their overall efficacy and demonstrated in vitro activity against *Y. pestis* (*55*,*76*,*83*), streptomycin and gentamicin remain first-line agents for treatment of bubonic plague. However, clinicians can consider alternative or dual therapy for patients with bubonic disease, particularly for those with large buboes. Surgical incision and drainage might be necessary if the bubo becomes suppurative (*2*).

If needed, additional tetracyclines can serve as within-class alternatives to the first-line agent doxycycline for primary bubonic and pharyngeal plague. Tetracycline is considered effective for treatment of bubonic plague on the basis of previous successful use in humans (*16*,*19*). Omadacycline, minocycline, and eravacycline are believed to be effective primarily by extrapolation; one study of omadacycline demonstrated in vitro activity against *Y. pestis* and in vivo efficacy for postexposure prophylaxis in mice (*87*).

Patients presenting with naturally acquired primary bubonic or pharyngeal plague who are stable and do not show signs of secondary septicemic or pneumonic plague can be treated with a single antimicrobial agent. Patients who present with primary bubonic or pharyngeal plague that has progressed to secondary pneumonic or septicemic plague should be treated according to the recommendations for pneumonic and septicemic plague (Table 1).

### Plague Meningitis

Plague meningitis is an uncommon clinical manifestation, reported to occur among 0.2%–7% of patients with naturally occurring plague (*12*). However, in the event of an intentional release of *Y. pestis* infecting many persons, numerous patients could develop this manifestation. Antimicrobials used to treat plague meningitis must demonstrate efficacy against *Y. pestis* and cross the blood-brain barrier to achieve sufficient levels within the cerebrospinal fluid. Chloramphenicol has a long history of successful use for treatment of plague, specifically plague meningitis (*12*,*52*,*88*,*89*). Because moxifloxacin and levofloxacin have robust activity against *Y. pestis* and excellent central nervous system penetration (*90*), these drugs should be effective for treatment of plague meningitis (Table 3). However, no human data are available on the use of moxifloxacin or levofloxacin for plague meningitis.

**TABLE 3:** Treatment of patients of all ages and pregnant women with plague meningitis

Population	Category	Antimicrobial* Class	Dosage
**Adults** **aged ≥18 yrs**	**First-line^†,§^**	Chloramphenicol^¶,^** Amphenicol	25 mg/kg every 6 hrs IV^††^ (maximum 1 g/dose)
		Levofloxacin *Fluoroquinolone*	750 mg every 24 hrs IV or PO
		Moxifloxacin *Fluoroquinolone*	400 mg every 24 hrs IV or PO
**Neonates, infants, and children aged ≤17 yrs (unless otherwise noted)**	**First-line^†,§^**	Chloramphenicol^¶,^** Amphenicol	Neonates aged ≤7 days: 25 mg/kg/dose every 24 hrs IV^††^ Neonates aged 8–28 days: 25 mg/kg/dose every 12 hrs IV^††^ Infants and children aged ≥29 days to ≤17 yrs: 25 mg/kg every 6 hrs IV^††^ (maximum 1 g/dose)
		Levofloxacin *Fluoroquinolone*^§§^	Neonates aged ≤28 days: 10 mg/kg/dose every 12 hrs IV Infants and children aged ≥29 days to ≤17 yrs: Body weight <50 kg: 8 mg/kg every 12 hrs IV or PO (maximum 250 mg/dose) Body weight ≥50 kg: 500–750 mg every 24 hrs IV or PO
		Moxifloxacin^¶¶^ *Fluoroquinolone*^§§^	Infants and children aged ≥3 mos to ≤23 mos: 6 mg/kg every 12 hrs IV or PO*** Children aged 2–5 yrs: 5 mg/kg every 12 hrs IV or PO*** Children aged 6–11 yrs: 4 mg/kg every 12 hrs IV or PO*** Children and adolescents aged 12 to ≤17 yrs: Body weight <45 kg: 4 mg/kg every 12 hrs IV or PO*** Maximum dose for all children <45 kg: 200 mg/dose Body weight ≥45 kg: 400 mg every 24 hrs IV or PO***

**Abbreviations:** IV = intravenous; PO = per os.

**Note:** All oral antimicrobials recommended in these guidelines can be administered via alternative enteral routes (e.g., nasogastric tube and gastric tube) except for ciprofloxacin.

*Antimicrobials are not listed in order of preference within each category.

^†^ Dual therapy with chloramphenicol plus moxifloxacin or levofloxacin should be used for initial treatment of patients with plague who present with symptoms of meningitis. If chloramphenicol is not available, a nonfluoroquinolone first-line or alternative antimicrobial for treatment of septicemic plague can be substituted (Table 1). Recommended treatment duration is 10–14 days.

^§^ For patients with secondary plague meningitis, chloramphenicol should be added to the patient’s existing antimicrobial treatment regimen for plague. If chloramphenicol is not available, or clinicians would prefer to avoid using this drug in young children because of potential adverse effects, moxifloxacin or levofloxacin can be added to the patient’s existing treatment regimen instead. After chloramphenicol, moxifloxacin, or levofloxacin have been added, the entire regimen of antimicrobials the patient is receiving for plague should be continued for an additional 10 days.

^¶^ Not approved by the Food and Drug Administration (FDA) for treatment of plague. Chloramphenicol has been used off label for the treatment of naturally occurring plague (*17**,**19**,**88*). Large-scale distribution and use of these antimicrobials during a bioterrorism response might be under FDA-issued Emergency Use Authorization.

** At the time of publication, these antimicrobials might not be readily and consistently available in the United States because of limited production.

^††^ After clinical improvement, chloramphenicol can be reduced to a lower dose of 12.5 mg/kg every 6 hours in adults and given orally. Serum concentration monitoring should be performed when available, especially in children (Source: Tunkel AR, Hartman BJ, Kaplan SL, et al. Practice guidelines for bacterial meningitis. Clin Infect Dis 2004;39:1267–84).

^§§^ Data on use of fluoroquinolones in infants and young children are limited.

^¶¶^ Moxifloxacin is not FDA approved for use in children aged ≤17 years but has been used off label (*79*). Data on use in neonates and children aged ≤2 months are extremely limited; however, successful use in neonates has been reported (**Source:** Watt KM, Massaro MM, Smith B, Cohen-Wolkowiez M, Benjamin DK Jr, Laughon MM. Pharmacokinetics of moxifloxacin in an infant with *Mycoplasma hominis* meningitis. Pediatr Infect Dis J 2012;31:197–9). For children aged 12–17 years weighing ≥45 kg with risk factors for cardiac events, consider 200 mg twice daily to reduce risk for QT prolongation.

*** Although no commercial liquid formulation is available for moxifloxacin, hospitals and compounding retail pharmacies can use a published recipe to make a liquid suspension.

When possible, dual therapy with chloramphenicol and moxifloxacin or levofloxacin should be used for initial treatment of patients with plague who present with signs of meningitis (e.g., nuchal rigidity). If chloramphenicol is not available, a nonfluoroquinolone first-line or alternative antimicrobial for treatment of septicemic plague can be substituted (Table 1). For patients who develop secondary plague meningitis while already receiving antimicrobial therapy, chloramphenicol should be added to the patient’s existing antimicrobial treatment regimen for plague (Table 3). If chloramphenicol is not available, or clinicians would prefer to avoid using this drug in young children because of potential adverse effects, moxifloxacin or levofloxacin can be added to the patient’s existing treatment regimen instead. After chloramphenicol, moxifloxacin, or levofloxacin have been added, the entire regimen of antimicrobials the patient is receiving for plague should be continued for an additional 10 days.

### Personal Protective Equipment

Standard precautions should be used when caring for all patients with plague. Pneumonic plague can spread from infected patients to others by respiratory droplets during close (<6 feet), sustained contact (*2*,*9*). Therefore, droplet precautions should be used in addition to standard precautions when providing care to patients with suspected or confirmed pneumonic plague (*8*,*91*). Droplet precautions can be discontinued when patients with pneumonic plague have received antimicrobials for at least 48 hours and have shown clinical improvement with markedly decreased sputum production (*8*). Consistent with standard precautions, health care providers should wear a mask, eye protection, and face shield when performing procedures likely to generate sprays or splashes, such as bubo aspiration (*91*). Because no evidence exists of airborne transmission of *Y. pestis*, particulate filtering facepiece respirators such as N95 respirators are not necessary when providing routine care for patients with pneumonic plague; however, these might be considered as an added precaution for health care providers performing aerosol-generating procedures.

### Antimicrobial Pre- and Postexposure Prophylaxis for Adults and Children

Pre-exposure prophylaxis for first responders and health care providers who will care for patients with pneumonic plague is not considered necessary as long as standard and droplet precautions can be maintained. In cases of surgical mask shortages, patient overcrowding, poor ventilation in hospital wards, or other crisis situations, pre-exposure prophylaxis might be warranted if sufficient supplies of antimicrobials are available (Table 4). Potential risks for antimicrobial pre-exposure prophylaxis must be carefully considered. No data are available on optimal duration of pre-exposure prophylaxis; however, on the basis of pathophysiology of *Y. pestis* and animal studies, prophylaxis can be discontinued 48 hours after the last perceived exposure (*92*). Although potentially helpful in certain situations, pre-exposure prophylaxis should not replace essential protective measures for first responders and health care providers, such as wearing personal protective equipment and isolating patients with suspected pneumonic plague when possible.

**TABLE 4:** Pre- and postexposure prophylaxis for adults and children potentially exposed to *Yersinia pestis*

Population	Category	Antimicrobial*^,†^ *Class*	Dosage^§^
**Adults** **aged ≥18 yrs**	**First-line**	Ciprofloxacin *Fluoroquinolone*	500–750 mg every 12 hrs PO
		Levofloxacin *Fluoroquinolone*	500–750 mg every 24 hrs PO
		Moxifloxacin *Fluoroquinolone*	400 mg every 24 hrs PO
		Doxycycline *Tetracycline*	100 mg every 12 hrs PO
	**Alternatives**	Ofloxacin^¶,^**^,††^ *Fluoroquinolone*	400 mg every 12 hrs PO
		Gemifloxacin^¶^ *Fluoroquinolone*	320 mg every 24 hrs PO
		Tetracycline^¶^ *Tetracycline*	500 mg every 6 hrs PO
		Omadacycline^¶^ *Tetracycline*	300 mg every 24 hrs PO
		Minocycline^¶^ *Tetracycline*	100 mg every 12 hrs PO
		Trimethoprim- sulfamethoxazole^¶^ *Sulfonamide*	5 mg/kg (trimethoprim component) every 12 hrs PO
**Children** **aged ≥1 mos to ≤17 yrs (unless otherwise noted)**	**First-line**	Ciprofloxacin *Fluoroquinolone*^§§^	15 mg/kg every 12 hrs PO (maximum 750 mg/dose)
		Levofloxacin *Fluoroquinolone*^§§^	Body weight <50 kg: 8 mg/kg every 12 hrs PO (maximum 250 mg/dose) Body weight ≥50 kg: 500–750 mg every 24 hrs PO
		Doxycycline *Tetracycline*^§§^	Body weight <45 kg: 2.2 mg/kg every 12 hrs PO Body weight ≥45 kg: 100 mg every 12 hrs PO
	**Alternatives**	Moxifloxacin^¶¶^ *Fluoroquinolone*^§§^	Infants and children aged ≥3 mos to ≤23 mos: 6 mg/kg every 12 hrs PO*** Children aged 2–5 yrs: 5 mg/kg every 12 hrs PO*** Children aged 6–11 yrs: 4 mg/kg every 12 hrs PO*** Children and adolescents aged 12 to ≤17 yrs: Body weight <45 kg: 4 mg/kg every 12 hrs PO*** Maximum dose for all children <45 kg: 200 mg/dose Body weight ≥45 kg: 400 mg every 24 hrs PO***
		Ofloxacin^¶,^**^,††^ *Fluoroquinolone*^§§^	7.5 mg/kg every 12 hrs PO (maximum 400 mg/dose)
		Tetracycline^¶^ *Tetracycline*^§§^	10 mg/kg every 6 hrs PO (maximum 500 mg/dose)
		Minocycline^¶^ *Tetracycline*^§§^	2 mg/kg every 12 hrs PO (maximum 100 mg/dose)
		Trimethoprim-sulfamethoxazole^¶^ *Sulfonamide*	Infants and children aged ≥2 mos to ≤17 yrs: 5 mg/kg (trimethoprim component) every 12 hrs PO

**Abbreviations:** PO = per os.

**Note:** All oral antimicrobials recommended in these guidelines can be administered via alternative enteral routes (e.g., nasogastric tube and gastric tube) except for ciprofloxacin.

* Prophylaxis with a single antimicrobial class is recommended for potentially exposed persons following a case of naturally acquired infection or intentional release of *Yersinia pestis,* with targeting of drug choice if engineered resistance is detected in the aftermath of a bioterrorism attack.

^†^ Antimicrobials are not listed in order of preference within each category.

^§^ Pre-exposure prophylaxis can be discontinued 48 hours after the last perceived exposure. Recommended duration for postexposure prophylaxis is 7 days.

^¶^ Not approved by the Food and Drug Administration (FDA) for treatment of plague. In some instances, these antimicrobials have been used off label for the treatment of naturally occurring plague (*17**,**19**,**88*). Large-scale distribution and use of these antimicrobials during a bioterrorism response might be under FDA-issued Emergency Use Authorization.

** Additional fluoroquinolone alternatives, such as delafloxacin, also can be considered depending on drug availability.

^††^ Ofloxacin suspension for oral liquid administration is not available in the United States.

^§§^ Data on use of fluoroquinolones and tetracyclines in infants and young children are limited. Because of the risk for permanent tooth discoloration and tooth enamel hypoplasia, tetracycline and minocycline should only be used for children aged <8 years when other prophylaxis options have been exhausted.

^¶¶^ Moxifloxacin is not FDA approved for use in children aged ≤17 years but has been used off label (79). Data on use in neonates and children aged ≤2 months are extremely limited; however, successful use in neonates has been reported (**Source:** Watt KM, Massaro MM, Smith B, Cohen-Wolkowiez M, Benjamin DK Jr, Laughon MM. Pharmacokinetics of moxifloxacin in an infant with *Mycoplasma hominis* meningitis. Pediatr Infect Dis J 2012;31:197–9). For children aged 12–17 years weighing ≥45 kg with risk factors for cardiac events, consider 200 mg twice daily to reduce risk for QT prolongation.

*** Although no commercial liquid formulation is available for moxifloxacin, hospitals and compounding retail pharmacies can use a published recipe to make liquid suspension.

Postexposure prophylaxis should be considered for persons who had close (<6 feet), sustained contact with a patient with pneumonic plague and were not wearing adequate personal protective equipment (Table 4). Antimicrobial postexposure prophylaxis also can be considered for laboratory workers accidentally exposed to infectious materials and persons who had close (<6 feet) or direct contact with infected animals, such as veterinary staff, pet owners, and hunters. Postexposure prophylaxis should be given for 7 days.

In the event of an intentional release of *Y. pestis*, persons who were likely exposed should be provided with postexposure prophylaxis rapidly. Monotherapy is recommended for postexposure prophylaxis in the aftermath of an intentional release, with targeting of drug of choice as indicated if engineered resistance has been detected.

First responders and health care providers who followed standard and droplet precautions while caring for patients with pneumonic plague do not need to take postexposure prophylaxis. Similarly, laboratory workers who followed standard procedures while handling specimens from patients with plague and who were not exposed by other means need not take postexposure prophylaxis. Exposed persons can be placed on fever watch if desired.

### Antimicrobial Treatment and Prophylaxis of Special Populations

Special populations, including pregnant or lactating women, neonates, the elderly, immunocompromised, and obese patients, have unique considerations that should be addressed when recommending antimicrobial treatment and prophylaxis for plague. These populations exhibit physiologic differences that might influence their susceptibility to plague and severity of disease as well as their metabolism of and response to certain antimicrobial regimens.

#### Pregnant Women

Pregnant women experience physiologic changes during pregnancy, including alterations in immunity, pulmonary function, gastrointestinal motility and absorption, renal function, and cardiovascular output. These changes, along with increased plasma volume and variations in drug metabolism, should be considered when selecting the type, dose, frequency, and route of administration of antimicrobials recommended for treatment and prophylaxis of plague among pregnant women. Clinicians also should consider available safety data related to use during pregnancy when selecting antimicrobials; however, fetal safety concerns should not prevent access to rapid treatment or prophylaxis for pregnant women during a plague outbreak.

Data on the severity of and antimicrobial treatment for *Y. pestis* infection during pregnancy are limited. A recent systematic literature review identified 160 published cases of plague during pregnancy and found an overall high proportion of fatalities (67%) and pregnancy loss (74%) among those who did not receive antimicrobial treatment (*21*). As expected, antimicrobial treatment was associated with decreased maternal fatality and pregnancy loss (29% and 62%, respectively). In addition to death, pregnant women with plague also were at risk for preterm birth and hemorrhage (i.e., postpartum hemorrhage and hemorrhage into tissues discovered on autopsy). Maternal-fetal transmission of *Y. pestis* in the absence of maternal antimicrobial treatment appears possible, although the frequency at which this occurs is unknown (*21*). These data have substantial limitations, including potential biases resulting from reliance on case reports and often incomplete data elements. Nevertheless, these findings suggest pregnant women experience severe consequences of plague and warrant timely and effective treatment and prophylaxis, similar to nonpregnant women.

Because plague has a high case-fatality rate, CDC recommends antimicrobial treatment and prophylaxis be given to affected pregnant women when indicated, even if antimicrobial use by pregnant women carries some risks for adverse events to the fetus. Effectiveness of the antimicrobial(s) should be the primary driver of antimicrobial choice. When different antimicrobial classes are available, safety profiles of various antimicrobials can inform selection of those that maximize benefit to the pregnant woman while minimizing potential risk. Data are minimal on the effectiveness of antimicrobials recommended for treatment and prophylaxis of plague. In the systematic review cited previously, survival among pregnant women treated with streptomycin was 92% (12 of 13 pregnant women), whereas 73% (11 of 15 pregnant women) survived when treated with sulfonamides. Pregnant women also were treated with penicillins (n = 3; 67% survival), tetracyclines (n = 3; 67% survival), gentamicin (n = 1; 100% survival), and chloramphenicol (n = 1; 100% survival); however, interpretation of these data are limited by very small numbers.

A review of the safety of fluoroquinolones during pregnancy did not find evidence of an association between maternal fluoroquinolone exposure and pregnancy loss or birth defects (*93*). A separate systematic review examined the safety of nonfluoroquinolone antimicrobials used for the treatment of plague and found that several were potentially associated with adverse maternal or fetal outcomes (*48*). Consequently, although the risks for plague underscore the need for antimicrobial treatment of pregnant women to approximate recommendations for the nonpregnant population, several modifications are warranted in settings where there are no antimicrobial shortages.

For treatment of all forms of plague, gentamicin is preferred over streptomycin because of the greater risk for irreversible fetal ototoxicity documented with streptomycin use during pregnancy (Tables 5 and 6). For aminoglycosides, clinicians should check drug concentrations as appropriate and adjust dose and duration accordingly because the increased plasma volume and increased glomerular filtration rate observed in pregnancy might decrease the drug exposure. Similarly, both ciprofloxacin and levofloxacin are preferred over moxifloxacin because of the lack of safety and efficacy data available on moxifloxacin use in pregnant women. However, if neither ciprofloxacin nor levofloxacin are available, moxifloxacin should be used when indicated for treatment or prophylaxis to prevent morbidity and mortality. Because of the increased renal clearance of ciprofloxacin during pregnancy, more frequent dosing is recommended (Table 5). The risks for aminoglycosides and fluoroquinolones might vary by trimester of pregnancy; however, additional data are needed to further characterize these differential risks.

**TABLE 5:** Treatment of pregnant women with pneumonic, septicemic, bubonic, or pharyngeal plague

Category	Antimicrobial*^,†^ *Class*	Dosage^§,¶^
**First-line: Gentamicin *plus* either ciprofloxacin or levofloxacin**	Ciprofloxacin *Fluoroquinolone*	400 mg every 8 hrs IV or 500 mg every 8 hrs PO
	Levofloxacin *Fluoroquinolone*	750 mg every 24 hrs IV or PO
	Gentamicin** Aminoglycoside	5 mg/kg every 24 hrs IV or IM
**Alternatives**	Moxifloxacin *Fluoroquinolone*	400 mg every 24 hrs IV or PO
	Ofloxacin**^,††^ *Fluoroquinolone*	400 mg every 12 hrs PO
	Streptomycin^§§^ *Aminoglycoside*	1 g every 12 hrs IV or IM
	Amikacin** Aminoglycoside	15–20 mg/kg every 24 hrs IV or IM
	Tobramycin** Aminoglycoside	5–7 mg/kg every 24 hrs IV or IM
	Plazomicin**^,§§^ *Aminoglycoside*	15 mg/kg every 24 hrs IV
	Doxycycline *Tetracycline*	200 mg loading dose IV, then 100 mg every 12 hrs IV or PO or 200 mg every 24 hrs IV
	Chloramphenicol**^,§§^ *Amphenicol*	12.5–25 mg/kg every 6 hrs IV^¶¶^ (maximum 1 g/dose)
	Trimethoprim-sulfamethoxazole** Sulfonamide	5 mg/kg (trimethoprim component) every 8 hrs IV or PO

**Abbreviations:** IM = intramuscular; IV = intravenous; PO = per os.

**Note:** All oral antimicrobials recommended in these guidelines can be administered via alternative enteral routes (e.g., nasogastric tube and gastric tube) except for ciprofloxacin.

* Dual therapy with distinct classes of antimicrobials is recommended for treatment of plague in pregnant women caused by naturally acquired infection or intentional release of *Yersinia pestis*.

^†^ Antimicrobials are not listed in order of preference within each category.

^§^ Recommended treatment duration is 10–14 days.

^¶^ Parenteral administration is preferred route for all antimicrobials initially, when applicable.

** Not approved by the Food and Drug Administration (FDA) for treatment of plague. In some instances, these antimicrobials have been used off label for the treatment of naturally occurring plague (*17**,**19**,**88*). Large-scale distribution and use of these antimicrobials during a bioterrorism response might be under FDA-issued Emergency Use Authorization.

^††^ Additional fluoroquinolone alternatives, such as gemifloxacin and delafloxacin, also can be considered depending on drug availability.

^§§^ At the time of publication, these antimicrobials might not be readily and consistently available in the United States because of limited production.

^¶¶^ The lower end of the chloramphenicol dosing range (12.5 mg/kg every 6 hours) is sufficient for treatment of plague in most cases. Severe infections might require increased dosing, but these doses should be decreased as soon as feasible (**Sources:** Chloramphenicol sodium succinate [Package insert]. Lake Zurich, IL: Fresenius Kabl, LLC; 2019. Chloromycetin sodium succinate [Package insert]. Bristol, TN: Monarch Pharmaceuticals, LLC; 2004). Serum concentration monitoring should be performed when available.

**TABLE 6:** Pre- and postexposure prophylaxis for pregnant women potentially exposed to *Yersinia pestis*

Category	Antimicrobial*^,†^ *Class*	Dosage^§^
**First-line**	Ciprofloxacin *Fluoroquinolone*	500 mg every 8 hrs PO or 750 mg every 12 hrs PO
	Levofloxacin *Fluoroquinolone*	750 mg every 24 hrs PO
**Alternatives**	Moxifloxacin *Fluoroquinolone*	400 mg every 24 hrs PO
	Ofloxacin^¶,^** Fluoroquinolone	400 mg every 12 hrs PO
	Tetracycline^¶,^**^††^** *Tetracycline*	500 mg every 6 hrs PO
	Doxycycline *Tetracycline*	100 mg every 12 hrs PO
	Minocycline^¶,^**^††^** *Tetracycline*	200 mg loading dose, then 100 mg every 12 hrs PO
	Trimethoprim-sulfamethoxazole^¶^ *Sulfonamide *	5 mg/kg (trimethoprim component) every 12 hrs PO

**Abbreviations:** PO = per os.

**Note:** All oral antimicrobials recommended in these guidelines can be administered via alternative enteral routes (e.g., nasogastric tube and gastric tube) except for ciprofloxacin.

* Prophylaxis with a single antimicrobial class is recommended for potentially exposed pregnant women following a case of naturally acquired infection or intentional release of *Yersinia pestis,* with targeting of drug choice if engineered resistance is detected in the aftermath of a bioterrorism attack.

^†^ Antimicrobials are not listed in order of preference within each category.

^§^ Pre-exposure prophylaxis can be discontinued 48 hours after the last perceived exposure. Recommended duration for postexposure prophylaxis is 7 days.

^¶^ Not approved by the Food and Drug Administration (FDA) for treatment of plague. In some instances, these antimicrobials have been used off label for the treatment of naturally occurring plague (*17**,**19**,**88*). Large-scale distribution and use of these antimicrobials during a bioterrorism response might be under FDA-issued Emergency Use Authorization.

** Additional fluoroquinolone alternatives, such as gemifloxacin and delafloxacin, also can be considered depending on drug availability.

^††^ In utero exposure can lead to permanent discoloration of developing teeth in the fetus. This is more likely to occur following repeated or long-term exposure.

Because of the potential for high maternal morbidity and mortality from plague, the risk for perinatal transmission, and the lack of data on pharmacokinetics of various antimicrobials among pregnant women, dual therapy with distinct classes of antimicrobials is recommended for treatment of plague in pregnant women caused by either naturally acquired infection or intentional release of *Y. pestis*. Specifically, gentamicin combined with either ciprofloxacin or levofloxacin is the preferred regimen. Parenteral administration, when applicable, is the preferred route for initial plague treatment because of the frequent inability of pregnant women to tolerate oral medications and the decreased GI absorption and motility that commonly occur during pregnancy. For pregnant women with obesity, additional dosing recommendations are available (see Persons Who Are Obese or Underweight).

For pregnant women with secondary plague meningitis, chloramphenicol should be added to the existing antimicrobial treatment regimen for all trimesters of pregnancy because it penetrates the blood-brain barrier and is the standard of care for nonpregnant patients. Although chloramphenicol carries a potential risk for serious blood dyscrasias and gray baby syndrome when given to neonates, occurrence resulting from in utero exposure is exceedingly rare (*41*).

To facilitate parenteral administration of antimicrobials and monitoring for preterm labor and maternal hemorrhage, hospitalization of pregnant women should be considered, particularly for those with pneumonic plague and those in the second and third trimester. As with other severe systemic infections, preterm labor might occur in pregnant women with plague. Administration of corticosteroids to pregnant women experiencing preterm labor to promote fetal lung maturity might be considered, in accordance with ACOG guidelines (*94*) and as deemed appropriate by the clinical care team. In addition to the antimicrobial recommendations included in these guidelines, standard guidelines for maternal sepsis should be followed when managing plague in pregnant women with associated signs of sepsis (*95*).

Pre- and postexposure prophylaxis recommendations for pregnant women reflect those for the nonpregnant population, with the exception that moxifloxacin and doxycycline should be considered alternatives rather than first-line antimicrobials (Table 7). Monotherapy for postexposure prophylaxis is preferred for management of exposures to *Y. pestis*., with targeting of drug choice as indicated if engineered resistance is detected in the aftermath of a bioterrorism attack. Enhanced antepartum fetal monitoring of asymptomatic pregnant women is not needed on the basis of potential exposure to *Y. pestis*.

**TABLE 7:** Treatment of neonates aged ≤28 days with pneumonic or septicemic plague

Category	Antimicrobial*^,†^ *Class*	Dosage^§^
**First-line**	Gentamicin^¶^ *Aminoglycoside*	Neonates aged ≤7 days: 4 mg/kg every 24 hrs IM or IV Neonates aged 8–28 days: 5 mg/kg every 24 hrs IM or IV
	Ciprofloxacin Fluoroquinolone**	10 mg/kg every 8 or 12 hrs IV
	Levofloxacin Fluoroquinolone**	10 mg/kg every 12 hrs IV
	Streptomycin^††^ *Aminoglycoside*	15 mg/kg every 12 hrs IV^§§^ or IM
**Alternatives**	Tobramycin^¶^ *Aminoglycoside*	Neonates aged ≤7 days: 4 mg/kg every 24 hrs IM or IV Neonates aged 8–28 days: 5 mg/kg every 24 hrs IM or IV
	Amikacin^¶^ *Aminoglycoside*	Neonates aged ≤7 days: 15 mg/kg every 24 hrs IM or IV Neonates aged 8–28 days: 17.5 mg/kg every 24 hrs IM or IV
	Chloramphenicol^¶,††^ *Amphenicol*	Neonates aged ≤14 days: 6.25 mg/kg every 6 hrs IM or IV Neonates aged 15–28 days: 12.5 mg/kg every 6 hrs IM or IV^¶¶^
	Doxycycline Tetracycline**	4.4 mg/kg loading dose, then 2.2 mg/kg every 12 hrs IV

**Abbreviations:** IM = intramuscular; IV = intravenous; PO = per os.

**Note:** All oral antimicrobials recommended in these guidelines can be administered via alternative enteral routes (e.g., nasogastric tube and gastric tube) except for ciprofloxacin. Additional considerations for treatment and postexposure prophylaxis of neonates, depending on the clinical status of both neonate and mother, are included in Supplementary Appendix 2 (https://stacks.cdc.gov/view/cdc/107427).

* Dual therapy with two distinct classes of antimicrobials should be used for initial treatment of neonates with severe pneumonic or septicemic plague and neonates infected after intentional release of *Yersinia pestis*.

^†^ Antimicrobials are not listed in order of preference within each category.

^§^ Recommended treatment duration is 10–14 days.

^¶^ Not approved by the Food and Drug Administration (FDA) for treatment of plague. In some instances, these antimicrobials have been used off label for the treatment of naturally occurring plague (*17**,**19**,**88*). Large-scale distribution and use of these antimicrobials during a bioterrorism response might be under FDA-issued Emergency Use Authorization.

** Data on use of fluoroquinolones and doxycycline in neonates are extremely limited; however, successful use of these antimicrobials in neonates has been reported (**Sources:** Kaguelidou F, Turner MA, Choonara I, Jacqz-Aigrain E. Ciprofloxacin Use in Neonates. Pediatr Infect Dis J 2011;30:e29–e37. Newby BD, Timberlake KE, Lepp LM, Mihic T, Dersch-Mills DA. Levofloxacin use in the neonate: A case series. J Pediatr Pharmacol Ther 2017;22:304–13. Forti G, Benincori C. Doxycycline and the teeth. Lancet 1969;1:782).

^††^ At the time of publication, these antimicrobials might not be readily and consistently available in the United States because of limited production.

^§§^ The IV formulation of streptomycin is not approved by the FDA; however, the IM formulation of streptomycin has been given intravenously as an off-label use (**Sources:** Morris JT, Cooper RH. Intravenous streptomycin: a useful route of administration. Clin Infect Dis 1994;19:1150–1. Pérez Tanoira R, Sánchez-Patán F, Jiménez Girón A. et al. Tolerance and safety of intravenous streptomycin therapy in patients with tuberculosis. Infection 2014;42:597–8).

^¶¶^ **Sources:** Chloramphenicol sodium succinate [Package insert]. Lake Zurich, IL: Fresenius Kabl, LLC; 2019. Chloromycetin sodium succinate [Package insert]. Bristol, TN: Monarch Pharmaceuticals, LLC; 2004. Serum concentration monitoring should be performed when available.

Some studies have identified an association between trimethoprim-sulfamethoxazole exposure in the first trimester among pregnant women not taking folic acid supplements and the development of fetal neural tube defects (*48*). However, this drug is used regularly during pregnancy. In the United States, all women capable of becoming pregnant are encouraged to consume at least 400 *μ*g of folic acid daily from supplements, fortified foods, or both, in addition to folate-rich foods from a varied diet, to reduce the risk for neural tube defects during early pregnancy (*96*). For women in the United States with known or suspected pregnancy and *Y. pestis* infection or exposure, the risk-benefit profile favors the use of trimethoprim-sulfamethoxazole during any trimester. For pregnant women prescribed trimethoprim-sulfamethoxazole in the third trimester, clinicians caring for the infant after delivery should be informed of this drug exposure so that they are aware of the risk for hyperbilirubinemia.

#### Neonates

Plague in neonates has rarely been documented. One report described a 7-day-old female who presented with fever and listlessness; 2 days earlier a red macule was observed on her knee, which was assumed to be the route of exposure via flea bite. She had a “septic appearance,” questionably full fontanel, and no lymphadenopathy. She recovered after treatment with ampicillin, gentamicin, and later streptomycin (*97*). Another report described a 1-month-old male with primary plague meningitis who presented with fever, convulsions, bulging fontanel, and no lymphadenopathy. He died on the 17th day of illness despite sulfathiazole therapy; autopsy revealed thick fibrino-purulent exudate covering the surface of the brain and spinal cord (*98*). It is unknown whether neonates with plague have distinct clinical manifestations compared with the general pediatric population.

Treatment should be initiated as soon as possible for symptomatic neonates who have been infected with *Y. pestis* before, during, or after birth (Tables 7 and 8). For these recommendations, neonates are defined as full-term infants aged ≤28 days or preterm infants 37–44 weeks postmenstrual age. IM administration should be used only if IV administration is not possible because of lack of IV access or resource constraints. Ofloxacin is not included as a treatment alternative because it is only available as an oral formulation. However, if first-line and alternative treatment options have been exhausted, clinicians can consider using ofloxacin as a last resort. Plazomicin and moxifloxacin have not been evaluated for use in neonates but could also be considered if other antimicrobials are not available. In cases of diagnostic uncertainty, neonates with possible plague versus nonplague sepsis can be treated with ampicillin and gentamicin, which would cover both *Y. pestis* and other pathogens that commonly cause sepsis among neonates.

**TABLE 8:** Treatment of neonates aged ≤28 days with bubonic or pharyngeal plague

Category	Antimicrobial*^,†^ *Class*	Dosage^§^
**First-line**	Gentamicin^¶^ *Aminoglycoside*	Neonates aged ≤7 days: 4 mg/kg every 24 hrs IM or IV Neonates aged 8–28 days: 5 mg/kg every 24 hrs IM or IV
	Ciprofloxacin Fluoroquinolone**	10 mg/kg every 8 or 12 hrs IV
	Levofloxacin Fluoroquinolone**	10 mg/kg every 12 hrs IV
	Streptomycin^††^ *Aminoglycoside*	15 mg/kg every 12 hrs IV^§§^ or IM
	Doxycycline Tetracycline**	4.4 mg/kg loading dose, then 2.2 mg/kg every 12 hrs IV
**Alternatives**	Tobramycin^¶^ *Aminoglycoside*	Neonates aged ≤7 days: 4 mg/kg every 24 hrs IM or IV Neonates aged 8–28 days: 5 mg/kg every 24 hrs IM or IV
	Amikacin^¶^ *Aminoglycoside*	Neonates aged ≤7 days: 15 mg/kg every 24 hrs IM or IV Neonates aged 8–28 days: 17.5 mg/kg every 24 hrs IM or IV
	Chloramphenicol^¶,††^ *Amphenicol*	Neonates aged ≤14 days: 6.25 mg/kg every 6 hrs IM or IV Neonates aged 15–28 days: 12.5 mg/kg every 6 hrs IM or IV^¶¶^

**Abbreviations:** IM = intramuscular; IV = intravenous.

**Note:** All oral antimicrobials recommended in these guidelines can be administered via alternative enteral routes (e.g., nasogastric tube and gastric tube) except for ciprofloxacin. Additional considerations for treatment and postexposure prophylaxis of neonates, depending on the clinical status of both neonate and mother, are included in Supplementary Appendix 2 (https://stacks.cdc.gov/view/cdc/107427).

* Dual therapy with two distinct classes of antimicrobials should be used for initial treatment of neonates infected after intentional release of *Yersinia pestis*.

^†^ Antimicrobials are not listed in order of preference within each category.

^§^ Recommended treatment duration is 10–14 days.

^¶^ Not approved by the Food and Drug Administration (FDA) for treatment of plague. In some instances, these antimicrobials have been used off label for the treatment of naturally occurring plague (*17**,**19**,**88*). Large-scale distribution and use of these antimicrobials during a bioterrorism response might be under FDA-issued Emergency Use Authorization.

** Data on use of fluoroquinolones and doxycycline in neonates are extremely limited; however, successful use of these antimicrobials in neonates has been reported (**Sources:** Kaguelidou F, Turner MA, Choonara I, Jacqz-Aigrain E. Ciprofloxacin use in neonates. Pediatr Infect Dis J 2011;30:e29–e37. Newby BD, Timberlake KE, Lepp LM, Mihic T, Dersch-Mills DA. Levofloxacin Use in the Neonate: A case series. J Pediatr Pharmacol Ther 2017;22:304–13. Forti G, Benincori C. Doxycycline and the teeth. Lancet 1969;1:782).

^††^ At the time of publication, these antimicrobials might not be readily and consistently available in the United States because of limited production.

^§§^ The IV formulation of streptomycin is not approved by FDA; however, the IM formulation of streptomycin has been given intravenously as an off-label use (**Sources:** Morris JT, Cooper RH. Intravenous streptomycin: a useful route of administration. Clin Infect Dis 1994;19:1150–1. Pérez Tanoira R, Sánchez-Patán F, Jiménez Girón A. et al. Tolerance and safety of intravenous streptomycin therapy in patients with tuberculosis. Infection 2014;42:597–8).

^¶¶^ **Sources:** Chloramphenicol sodium succinate [Package insert]. Lake Zurich, IL: Fresenius Kabl, LLC; 2019. Chloromycetin sodium succinate [Package insert]. Bristol, TN: Monarch Pharmaceuticals, LLC; 2004. Serum concentration monitoring should be performed when available.

Although chloramphenicol is generally contraindicated for infants aged <6 months because of the risk for serious blood dyscrasias or circulatory collapse (gray baby syndrome), it is still an appropriate option for treatment of plague meningitis in neonates because of the severity of this clinical presentation. Levofloxacin also can be used for treatment of plague meningitis (Table 3).

Postexposure prophylaxis should be given to all neonates exposed postnatally to *Y. pestis* (Table 9). Prophylaxis also should be considered for asymptomatic neonates born to mothers infected with *Y. pestis* during the last 7 days of pregnancy if the mother was untreated or treated <48 hours before delivery (Supplementary Appendix 2, https://stacks.cdc.gov/view/cdc/107427). Careful observation, rather than antimicrobial prophylaxis, is acceptable for asymptomatic neonates born to infected but appropriately treated mothers and for asymptomatic neonates born to mothers exposed to *Y. pestis* (Supplementary Appendix 2, https://stacks.cdc.gov/view/cdc/107427).

**TABLE 9:** Postexposure prophylaxis for neonates aged ≤28 days potentially exposed to *Yersinia pestis*

Category	Antimicrobial*^,†^ *Class*	Dosage^§^
**First-line**	Ciprofloxacin *Fluoroquinolone*^¶^	15 mg/kg every 12 hrs PO
	Levofloxacin *Fluoroquinolone*^¶^	10 mg/kg every 12 hrs PO
	Doxycycline *Tetracycline*^¶^	2.2 mg/kg every 12 hrs PO
**Alternatives**	Gentamicin** Aminoglycoside	Neonates aged ≤7 days: 4 mg/kg every 24 hrs IM or IV Neonates aged 8–28 days: 5 mg/kg every 24 hrs IM or IV
	Ofloxacin**^,††^ *Fluoroquinolone*^¶^	7.5 mg/kg every 12 hrs PO

**Abbreviations:** IM = intramuscular; IV = intravenous; PO = per os.

**Note:** All oral antimicrobials recommended in these guidelines can be administered via alternative enteral routes (e.g., nasogastric tube and gastric tube) except for ciprofloxacin. Additional considerations for treatment and postexposure prophylaxis of neonates, depending on the clinical status of both neonate and mother, are included in Supplementary Appendix 2 (https://stacks.cdc.gov/view/cdc/107427).

* Antimicrobials are not listed in order of preference within each category.

^†^ Postexposure prophylaxis with a single antimicrobial agent is recommended for potentially exposed neonates following a case of naturally acquired infection or intentional release of *Yersinia Pestis*, with targeting of drug of choice if engineered resistance is detected in the aftermath of a bioterrorism attack. Postexposure prophylaxis should be given to neonates orally when possible, unless the neonate is hospitalized and has existing intravenous access. For neonates with highly concerning exposure to *Y. pestis* who cannot take medications orally, IV or IM formulations of the drugs listed in this table can be given.

^§^ Recommended postexposure prophylaxis duration is 7 days.

^¶^ Data on use of fluoroquinolones and doxycycline in neonates are extremely limited; however, successful use of these antimicrobials in neonates has been reported (**Sources:** Kaguelidou F, Turner MA, Choonara I, Jacqz-Aigrain E. Ciprofloxacin use in neonates. Pediatr Infect Dis J 2011;30:e29–e37. Newby BD, Timberlake KE, Lepp LM, Mihic T, Dersch-Mills DA. Levofloxacin Use in the Neonate: A case series. J Pediatr Pharmacol Ther 2017;22:304–13. Forti G, Benincori C. Doxycycline and the teeth. Lancet 1969;1:782).

** Not approved by the Food and Drug Administration (FDA) for prophylaxis of plague. In some instances, these antimicrobials have been used off label for the prophylaxis of naturally occurring plague (*17**,**19**,**88*). Large-scale distribution and use of these antimicrobials during a bioterrorism response might be under FDA-issued Emergency Use Authorization.

^††^ Ofloxacin suspension for oral liquid administration is not available in the United States.

Three important factors should be considered when selecting antimicrobials for treatment and prophylaxis of plague among neonates. First, many medications must be given to neonates via the IV route to ensure that the full desired dose is successfully administered. Some neonates regularly experience gastresophageal reflux (spitting up), making it difficult to administer a full medication dose orally. Moreover, neonates regularly ingest breast milk or formula, which have substantial amounts of calcium and other minerals that can inhibit absorption of some antimicrobials. For example, bioavailability of oral fluoroquinolones and tetracyclines is reduced by ingestion of milk (*99*,*100*). Although ofloxacin has not been examined directly, reduced bioavailability with co-ingestion with milk is possible because it is a fluoroquinolone. Second, many antimicrobials have not been specifically evaluated or approved for use in neonates. Third, some antimicrobials also carry specific or enhanced risks for neonates, such as bilirubin displacement and the potential for kernicterus associated with sulfonamides. Antimicrobial use in neonates also can lead to general adverse reactions such as disruption of the gut microbiome (*101*,*102*).

#### Lactating Mothers and Breastfeeding Infants

Breastfeeding has many benefits including maternal-infant bonding; ideal nutrition for the infant; and boosting of the immune system via immunoglobulins, cytokines, probiotic bacteria, and other protective immunologic factors contained in breast milk (*103*,*104*). Therefore, any potential risk related to transmission of infectious pathogens via breast milk must be balanced against the protective effects of breastfeeding.

Although no studies have directly assessed the presence of *Y. pestis* in breast milk of infected mothers, no reports exist of suspected *Y. pestis* transmission from mother to child through breast milk. Therefore, the risk for *Y. pestis* transmission through breast milk is believed to be low.

Mothers with bubonic or septicemic plague and mothers taking antimicrobial prophylaxis after exposure to *Y. pestis* can continue to breastfeed their infants if able. A mother with pneumonic plague can continue to breastfeed if she is receiving antimicrobial treatment and her infant is receiving antimicrobial treatment or postexposure prophylaxis for *Y. pestis*. Because of the risk for person-to-person transmission of pneumonic plague, for infants not receiving antimicrobial treatment or prophylaxis for *Y. pestis*, mothers with pneumonic plague should avoid direct breastfeeding until they have received antimicrobial treatment for ≥48 hours and demonstrated clinical improvement. Regular expression of breast milk via hand or mechanical pump by the mother should be encouraged and supported during this time; expressed breast milk can be given to the infant. Some mothers might need lactation support to promote milk supply, support the breastfeeding relationship, and prevent breast infections.

Clinicians selecting antimicrobials for lactating mothers who require treatment or prophylaxis for *Y. pestis* should take into account transmission of the drug or metabolites to the infant via breast milk and potential consequences to the infant. Apart from chloramphenicol, the majority of antimicrobials recommended for *Y. pestis* treatment or prophylaxis produce low concentrations in breast milk and have an acceptable safety profile (Supplementary Appendix 3, https://stacks.cdc.gov/view/cdc/107427).

Fluoroquinolones are present in breast milk in quantities far below the usual pediatric dosage for these medications. Among the fluoroquinolones, the best studied are ciprofloxacin and ofloxacin, which are present in breast milk at concentrations two orders of magnitude lower than a typical therapeutic infant dose (*105*,*106*). In addition, absorption of fluoroquinolones from breast milk is expected to be reduced because of the high concentration of calcium in breast milk (*102*,*105*,*106*). The theoretical risk for complications from fluoroquinolones can be reduced by timing breastfeeding to correspond with the time of lowest concentration of the drug in breast milk; for ciprofloxacin, this is 3–4 hours after each dose, and for ofloxacin 4–6 hours (*105*,*106*).

Aminoglycosides are poorly absorbed by the gastrointestinal tract and, like fluoroquinolones, are only present in breast milk in very low quantities. Gentamicin, amikacin, tobramycin, and streptomycin are detected in concentrations of 0.4–5.2 mg/L in breast milk (*107*).

Tetracyclines also are present only in very low levels in breast milk, and the calcium in breast milk might inhibit absorption by infants (*108*,*109*). The best studied antimicrobials in this class is tetracycline. One study of mothers on a steady state dose found that tetracycline reached a peak breast milk concentration of only 2.58 mg/L and was undetectable in the serum of their breastfeeding infants (*110*). Nevertheless, tetracycline should only be prescribed for short-term use with avoidance of repeated courses. Doxycycline is now considered acceptable for short-term use even in children aged <8 years, and because of the low concentrations of all tetracyclines found in breast milk, significant adverse reactions are unlikely (*110*). Minocycline has been associated with black discoloration of breast milk believed to be caused by iron pigment deposition in macrophages (*111*,*112*). Although this is not likely to pose a risk for mothers or infants, this effect might be worrisome to patients and might be a reason to choose another agent when available.

Trimethoprim-sulfamethoxazole is found in very low levels in breast milk, with infant drug levels in breast milk an order of magnitude lower than the therapeutic dose (*113*). There is substantial clinical experience in using trimethoprim-sulfamethoxazole near-term and during breastfeeding in mothers living with HIV, and an extensive review has found no adverse events in this population (*114*). Nevertheless, a theoretical risk for bilirubin displacement exists in susceptible infants, including those aged ≤28 days and those with pre-existing jaundice, prematurity, glucose-6-phosphate deficiency, or other vulnerabilities (*115*). Alternative agents should be selected for these populations, if available.

Chloramphenicol has the potential for serious adverse reactions in infants, and other drugs should be used preferentially for breastfeeding mothers (*116*,*117*). Chloramphenicol is expressed in breast milk at an average maximum concentration of 6.1 mg/L, and the drug has high oral absorption (*118*). Gray baby syndrome has only occurred in children receiving >200 mg/day (*117*). On the other hand, aplastic anemia is believed to be dose independent and is a theoretical risk; however, no cases of aplastic anemia have been reported to date among infants of nursing mothers receiving chloramphenicol. If a nursing mother must be treated with chloramphenicol, the infant should be monitored for gastrointestinal distress, adequacy of nursing, and blood dyscrasias (using a complete blood count with differential panel) (*116*).

Breastfeeding infants who are given antimicrobials and whose mothers also require treatment or prophylaxis for plague can receive antimicrobials via two sources: direct administration and indirectly via breast milk. This can lead to higher doses than intended of one antimicrobial or drug-drug interactions between two different antimicrobials. However, the risk for inadvertently administering a toxic dose of any antimicrobial via breast milk is low for every drug except chloramphenicol. Fluoroquinolones, aminoglycosides, tetracyclines, and trimethoprim-sulfamethoxazole are all excreted in breast milk in minute quantities relative to therapeutic dosing levels. Similarly, the risk for a dangerous drug-drug interaction also is low. Therefore, the antimicrobial regimen of the lactating mother typically should not influence antimicrobial selection for the breastfeeding infant.

#### Persons Who Are Elderly or Immunocompromised

Among 762 patients with plague summarized in a systematic literature review, 19 (2%) were aged ≥65 years. These elderly patients with plague had a 42% case fatality rate, more than twice the 19% case-fatality rate found for patients aged <65 years (*19*).

Survival rates for 19 patients aged ≥65 years who received antimicrobial monotherapy or combination therapy were 100% with chloramphenicol (n = 2), 80% with tetracyclines (n = 5), 80% with streptomycin (n = 5), 67% with fluoroquinolones (n = 3), 50% with gentamicin (n = 8), and 40% with sulfonamides (n = 5). However, interpretation is limited by small numbers of cases, combination therapy, and potential treatment bias (*19*).

Data on plague among immunocompromised patients are limited. One notable case of plague in a patient with potential immunocompromising factors occurred in 1992. A man aged 44 years who had undergone splenectomy and was receiving chemotherapy for idiopathic thrombocytopenic purpura (vinblastine sulfate and daily prednisone) was scratched by a *Y. pestis*-infected cat and developed bubonic plague. Symptoms included fever, nausea, vomiting, fatigue, an enlarged axillary lymph node, and swelling in the distal epitrochlear node. The patient recovered without complications after treatment with gentamicin and doxycycline (*89*). Eight additional patients with plague and underlying conditions (e.g., diabetes, renal insufficiency, histiocytosis X, alcoholism, and hepatitis B infection) also have been described (*119*–*126*), four of whom died (*120*–*122*,*126*), although the severity of these underlying conditions and whether they contributed to immunocompromise is unknown.

Treatment recommendations for patients who are elderly or immunocompromised do not differ from those for adults. However, treating clinicians should recognize the potential for decreases in glomerular filtration rate and presence of polypharmacy, with resultant drug-drug interactions, and adjust antimicrobials accordingly. Treating clinicians should carefully monitor patients with plague who are elderly or immunocompromised and extend therapy duration as needed on the basis of their clinical judgment.

For prophylaxis of elderly persons, clinicians should consider using doxycycline preferentially over fluoroquinolones because of the risks for QTc prolongation, neuropsychiatric disturbances including dizziness and imbalance, hypoglycemia, aortic rupture, and damage to connective tissues with fluoroquinolone use (*44*). Trimethoprim, alone or in combination with sulfamethoxazole, is associated with increased risk for acute kidney injury and hyperkalemia. This risk appears to be magnified when used concurrently with an angiotensin-converting enzyme inhibitor, angiotensin II receptor blocker, or potassium-sparing diuretic (*127*).

#### Persons Who Are Obese or Underweight

Body weight can affect both volume of distribution as well as metabolism and elimination of antimicrobials. In general, adjustments in dose are most important for drugs with a narrow therapeutic index. These adjustments are independent of those related to alterations in hepatic or renal function.

Aminoglycosides have a narrow therapeutic index and require therapeutic drug monitoring to avoid toxicity. For patients who are underweight (Body mass index [BMI] <18.5), initial dosing should be based on total body weight (TBW). For patients who are obese (BMI ≥30.0), initial dosing should be based on adjusted body weight (ABW), calculated as ideal body weight (IBW) plus 40% of excess body weight: ABW = IBW + ([TBW-IBW] x 0.4) (*128*). In addition, once daily dosing of aminoglycosides might not be appropriate for patients with severe obesity (BMI ≥40). Trimethoprim-sulfamethoxazole also should be dosed according to ABW in obese adults when prescribing doses >8 mg/kg/day (Supplementary Appendix 4, https://stacks.cdc.gov/view/cdc/107427) (*128*).

Ciprofloxacin should be dosed at the upper end of the dosing range for patients who are obese. No dosing adjustment is necessary for levofloxacin or moxifloxacin. Similarly, tetracyclines do not require dose adjustment for patients who are obese or underweight (*128*). Chloramphenicol should be dosed according to TBW in all cases, and serum concentration monitoring should be performed when available, especially in children (*129*).

For children, less specific dosing information is available. Evidence indicates that aminoglycosides can be dosed according to ABW for children who are obese (as for adults), whereas all other agents should be dosed according to TBW (*130*–*132*). In all cases, total dose for children should not exceed the maximum dose for an adult (*133*).

## Future Directions

Future efforts should attempt to address remaining gaps in the prevention, recognition, and clinical management of plague. Emergency preparedness should include planning for bioterrorism events that could exhaust U.S. antimicrobial, ventilator, and other supplies in clinical settings and in stockpiles. In terms of prevention, efforts to develop a plague vaccine effective against all forms of the disease should continue, and the need for scaling up such a vaccine for potential broad distribution should be anticipated. Previously available vaccines against *Y. pestis* have not demonstrated sufficient efficacy for pneumonic plague (*134*). Newer formulations of plague vaccines are in development or available for experimental use (*135*). Additional research to expand antimicrobial options, formulations and administration routes (e.g., inhaled gentamicin) (*136*), and adjunct therapies such as steroids also would be helpful. Development and application of algorithms capable of identifying high-risk patients and strategies for optimizing triage and resource allocation could play a crucial role in improving the ability to appropriately prepare for and respond to a mass-casualty event involving *Y. pestis*. As new research or other data become available regarding treatment and prophylaxis of *Y. pestis*, these guidelines will be revised and updated.

## Limitations

These guidelines are subject to at least three limitations. First, many of the recommendations are based on systematic review data derived from case reports and case series, both of which are widely recognized as low quality and biased sources of data. Large randomized controlled trials of antimicrobial safety and efficacy in humans with plague are notoriously challenging, because of resource constraints and the sporadic nature of plague outbreaks. Second, small numbers of patients in certain treatment groups hindered the ability to compare efficacy and survival rates between different antimicrobials. Third, nonhuman primate data considered as part of the guideline development process are informative but do not fully replicate the human experience.

## Conclusion

Plague has a high case-fatality rate but is treatable with antimicrobials and supportive care. Thus, early recognition of disease and administration of effective antimicrobials to treat plague are paramount to saving lives. In addition, persons exposed to *Y. pestis* can avoid illness if given effective antimicrobial prophylaxis in a timely manner.

Aminoglycosides and fluoroquinolones are the mainstays of antimicrobial treatment for plague. Depending on the clinical form of disease and the age and pregnancy status of the patient, tetracyclines, chloramphenicol, and trimethoprim-sulfamethoxazole also might be suitable antimicrobials for treatment. In the event of a bioterrorist attack using a strain of *Y. pestis* engineered for resistance, dual therapy with distinct classes of antimicrobials is recommended.

These guidelines provide recommendations for the antimicrobial treatment and prophylaxis of plague resulting from either naturally occurring transmission or a bioterrorism-related event. In addition to aiding health care providers caring for patients with plague, these recommendations can strengthen and support emergency response plans by local, state, and federal organizations.

## Footnotes

* 21st Century Cures Act, Pub. L. No. 114–255.

† Standards of Ethical Conduct for Employees of the Executive Branch, 5 CFR ^§^2635.
